# Strategy for Local Plant-Based Material Valorisation to Higher-Value Feed Stock for Piglets

**DOI:** 10.3390/ani12091092

**Published:** 2022-04-22

**Authors:** Sarunas Badaras, Modestas Ruzauskas, Romas Gruzauskas, Egle Zokaityte, Vytaute Starkute, Ernestas Mockus, Jolita Klementaviciute, Vadims Bartkevics, Laurynas Vadopalas, Dovile Klupsaite, Agila Dauksiene, Gintare Zokaityte, Ruta Mickiene, Elena Bartkiene

**Affiliations:** 1 Institute of Animal Rearing Technologies, Faculty of Animal Sciences, Lithuanian University of Health Sciences, Mickeviciaus Str. 9, LT-44307 Kaunas, Lithuania; sarunas.badaras@lsmuni.lt (S.B.); egle.zokaityte@lsmuni.lt (E.Z.); vytaute.starkute@lsmuni.lt (V.S.); ernestas.mockus@lsmuni.lt (E.M.); jolita.klementaviciute@lsmuni.lt (J.K.) laurynas.vadopalas@lsmu.lt (L.V.); dovile.klupsaite@lsmuni.lt (D.K.); agila.dauksiene@lsmuni.lt (A.D.); gintare.zokaityte@lsmuni.lt (G.Z.); 2 Institute of Microbiology and Virology, Faculty of Veterinary Medicine, Lithuanian University of Health Sciences, Mickeviciaus Str. 9, LT-44307 Kaunas, Lithuania; modestas.ruzauskas@lsmuni.lt; 3 Department of Anatomy and Physiology, Faculty of Veterinary Medicine, Lithuanian University of Health Sciences, Mickeviciaus Str. 9, LT-44307 Kaunas, Lithuania; 4 Department of Food Science and Technology, Kaunas University of Technology, Radvilenu Rd. 19, LT-50254 Kaunas, Lithuania; romas.gruzauskas@ktu.lt; 5 Department of Food Safety and Quality, Faculty of Veterinary Medicine, Lithuanian University of Health Sciences, Mickeviciaus Str. 9, LT-44307 Kaunas, Lithuania; 6 Institute of Food Safety, Animal Health and Environment BIOR, Lejupes ilea 3, LV-1076 Riga, Latvia; vadims.bartkevics@bior.gov.lv; 7 Instrumental Analysis Open Access Centre, Faculty of Natural Sciences, Vytautas Magnus University, Vileikos 8, LT-44404 Kaunas, Lithuania; ruta.mickiene@vdu.lt

**Keywords:** wheat bran, extrusion, fermentation, sugar beet pulp, piglets, microbiota

## Abstract

**Simple Summary:**

The aim of this study was to increase the sustainability of piglets farming by including valorised local cereal industry by-products as value-added feed material. In addition, dried sugar beet pulp, as local, high-energy, and nutritionally dense feed material for piglet feeding, was tested. The influence of the by-products on piglets’ blood parameters, faecal microbial and physico-chemical characteristics, and growth performance was evaluated. The control group was fed without by-product addition. A 41-day experiment was conducted using 300 (21-day-old) Large White/Norwegian Landrace piglets. Results showed that an extrusion and fermentation combination is a suitable strategy for wheat bran valorisation, as pre-treated wheat bran showed desirable antimicrobial and antifungal properties. Both treatments reduced the total enterobacteria and increased the lactic acid bacteria (LAB) count in piglets’ faeces. The consistency of the piglets’ faeces was within a physiological range throughout the whole experiment. It was established that the LAB count in piglets’ faeces is associated with specific volatile compounds’ formation (butanoic acid; butanoic acid, 3-methyl-; butyric acid (2-methyl-); pentanoic acid). Finally, the significantly higher body weight gain of the treatment groups could be associated with desirable changes in micro-organism populations in the piglets’ faeces, and the tested local feed material could be suggested for piglets’ nutrition.

**Abstract:**

In this study, a 41-day experiment was conducted using 300 (21-day-old) Large White/Norwegian Landrace piglets (100 piglets in each group). Three dietary treatments were compared: (i) a basal diet (C-I), (ii) a basal diet with the addition of extruded–fermented wheat bran (W_ex130/screwspeed25Lpa_) (TG-II), and (iii) a basal diet with the addition of dried sugar beet pulp (TG-III). Analyses of piglets’ blood parameters, faecal microbial and physico-chemical characteristics, and piglets’ growth performance were performed. It was found that the extrusion and fermentation combination led to an additional functional value of W_ex130/screwspeed25Lpa_, which showed desirable antimicrobial and antifungal properties in vitro (inhibited 5 out of 10 tested pathogenic strains and 3 out of 11 tested fungi). Both treatments reduced total enterobacteria and increased lactic acid bacteria counts in piglets’ faeces. The consistency of the piglets’ faeces (in all three groups) was within a physiological range throughout the whole experiment. Strong positive correlations were found between the LAB count in piglets’ faeces and butanoic acid; butanoic acid, 3-methyl-; butyric acid (2-methyl-); pentanoic acid. The treatment groups obtained a significantly higher body weight gain and average daily gain. Finally, substituting the piglets’ diet with W_ex130/screwspeed25Lpa_ and sugar beet pulp led to favourable changes in micro-organism populations in the piglets’ faeces as well as better growth performance.

## 1. Introduction

About 30 per cent of the planet’s ice-free terrestrial surface is used for livestock production [1]. Unfortunately, most of the livestock sector is organised in long market chains [2], which are associated with low sustainability and increasing climate change. Despite that, until recently, there were significant differences between rich and poor countries in animal-based product consumption; 17 and 33 per cent of kilocalorie and protein consumption, respectively, occurs from livestock sources [3]. Livestock sub-sector growth induces many effects (desirable and non-desirable) on the natural agricultural resource, public health, and economic growth [4]. However, there are some limits predicted for crops, such as the food and feed stock production sector, because of limited area expansion. In addition, in the near future, meeting the increases in demand for food, as well as the demand to reduce problems associated with climate change, will have some influence on livestock production systems over the coming years. In view of these challenges, livestock sector reorganisation from long to short market chains becomes very important, as well as the development of valorisation strategies for plant-based industry by-products for added-value feed stock.

Although wheat (*Triticum* spp.) is one of the most popular crops, until now, the outer layers (bran) have been used as a low-nutritional-value feed stock. To increase the effectiveness of wheat bran utilisation, pre-treatment technologies to improve the properties of these by-products were studied [5,6]. It was established that the combination of extrusion and fermentation technologies leads to safer (lower microbial contamination, lower mycotoxin and biogenic amine concentrations) added-value (lower pH, high viable lactic acid bacteria (LAB) count, higher concentration of free amino acids, etc.) stock, which could be applied to food as well as the feed industry. In addition, feeding experiments and clinical trials have shown the desirable effect of viable LAB on piglets’ intestinal microbiota [7,8,9]. LAB are known for their antagonistic effect against pathogenic bacteria as well as fungi [10]. From this point of view, they could be valuable ingredients in feed preparation and piglet nutrition.

Another food (refined sugar) industry by-product is sugar beet pulp. The total quantity of sugar produced annually from beet is, on average, the same as that produced from cane [11]. Sugarbeet processing is the production of saccharose from the above-mentioned plants. The main by-products of sugarbeet processing are molasses (MO) and pulp. Most of the MO produced is processed further to remove the remaining saccharose [12]. Around 20 million tonnes of sugar beet pulp are produced in Europe each year [13]. Sugar beet pulp has high concentrations of nitrogen-free leachate, crude fibre, and protein [14]. In addition, beet pulp is a soluble fibre source, shows good fermentable capacities, and could assist in preventing post-weaning diarrhoea, as well as improve intestinal morphology and growth performance in weaned pigs [15,16,17].

Despite the above-mentioned desirable characteristics, sugar processing by-products are not efficient enough in livestock production. Sugar beet could be a valuable material in piglet feeds, and such a conversion of by-products to animal-based production could be very beneficial.

In this study, we hypothesised that the local food industry by-products (valorised wheat bran and sugar beet pulp) could be included in piglets’ feeding as an added-value feed stock, which could lead to desirable changes in the microbial population of the piglets’ digesta, and the latter could be associated with changes in faecal volatile compounds, which could be used as chemical markers to indicate piglets’ health statuses.

Currently, there is an increasing demand to develop innovative means to monitor and, if necessary, correct animal health status. As a part of this, the microbiota and changes in the faecal volatile compound (VC) profile could be the first point for correcting a nutritional inbalance. Nutrition is related to changes in the faecal VC profile [18]. The effective functioning of the digestive system is an important factor in determining livestock welfare [19]. Up-to-date diagnostics of animal diseases are performed by causing animal stress; therefore, the development of non-invasive or minimally invasive measures, such as markers for gastrointestinal functionality identification, are promising tools that may reduce animal stress and increase productivity.

Finally, in this study, in addition to the newly developed strategy for local plant-based material (wheat bran) valorisation to a higher-value feed stock for piglets, the influence of three dietary treatments was compared. Despite the fact that sugar beet pulp is a well-known material for feed preparation, studies about the influence of this material on the microbial and VC profiles of piglets’ faeces are scarce. Additionally, valuable results could be obtained by comparing different treatments, which are based on feed materials composed of different dietary fibre compositions (wheat bran—source of non-soluble fibre, and sugar beet pulp—source of soluble fibre), and we hypothesised that these differences could be related to the differences in the microbiota and VC profiles of piglets’ faeces.

The aim of this study was to create a new strategy for piglet nutrition by including valorised local cereal industry by-products as an added-value feed material. In addition, sugar beet by-products (dried pulp), as local, high-energy, and nutritionally dense feed material for piglet feeding, were tested. The influence of both by-products on blood parameters, faecal microbial, and volatile compound profiles, as well as growth performance, was evaluated.

## 2. Materials and Methods

### 2.1. Cereal Industry By-Products and Sugar Beet Pulp Used for Piglet Feeding

Unprocessed and extruded wheat bran (WB) were obtained from the SME ‘Ustukiu malunas’ (Pasvalys, Lithuania). Extrusion parameters are given in Supplementary File S1. Four different treated WB samples were prepared: W_ex115_—xtruded at 115 °C with a screw speed of 16 rpm; W_ex130/screwspeed16_—extruded at 130 °C and 16 rpm; W_ex130/screwspeed20_—extruded at 130 °C and 20 rpm; W_ex130/screwspeed25_—extruded at 130 °C and 25 rpm. Non-extruded WB was used as a control (W_Con_—control WB sample).

Antimicrobial and antifungal properties possessing LAB strains *Lactobacillus casei* and *Lactobacillus paracasei* (obtained from the Lithuanian University of Health Sciences, Kaunas, Lithuania) [10] were used for fermentation of extruded and non-extruded WB. Parameters of fermentation applied for cereal industry by-product treatments are given in Supplementary File S1.

Finally, ten fermented WB samples were prepared: from non-extruded WB: W_ConLc_, W_ConLpa_; extruded at 115 °C with a screw speed of 16 rpm and fermented: W_ex115Lc_, W_ex115Lpa_; extruded at 130 °C and 16 rpm and fermented: W_ex130/screwspeed16Lc_, W_ex130/screwspeed16Lpa_; extruded at 130 °C and 20 rpm and fermented: W_ex130/screwspeed20Lc_, W_ex130/screwspeed20Lpa_; extruded at 130 °C and 25 rpm and fermented: W_ex130/screwspeed25Lc_, W_ex130/screwspeed25Lpa_.

The principal scheme of WB pre-treatment is given in Supplementary File S1. Physico-chemical and microbiological characteristics of the extruded and fermented WB were reported by Zokaityte et al. and Bartkiene et al. [5,6].

For piglet feeding (according to the lowest mycotoxin content [5,6]), W_ex130/screwspeed25Lpa_ samples were selected. In this study (in addition to previously reported physico-chemical parameters), antimicrobial and antifungal properties of the treated WB were evaluated.

Sugar beet pulp (dried) was obtained from the Imlitex Agro (Kaunas, Lithuania) company. The composition of sugar beet pulp was as follows: moisture—12.00%, crude protein—10.41%, crude fat—0.85%, crude fibre—17.82%, calcium—1.34%, phosphorus—0.12%.

### 2.2. Evaluation of Cereal Industry By-Products’ Antimicrobial and Antifungal Activity

Antimicrobial activity of WB against a variety of pathogenic and opportunistic bacterial strains (*E. coli, Bacillus pseudomycoides*, *Hafnia alvei*, *Enterococcus durans*, *Aeromonas veronii*, *Salmonella enterica Infantis*, *Staphylococcus aureus*, *Cronobacter sakazakii*, *Kluyvera cryocrescens*, and *Acinetobacter johnsonii*) was assessed by measuring the diameter of inhibition zones (DIZ, mm) in agar well diffusion assays, as described by Bartkiene et al. [10].

The antifungal activities of the cereal by-products were determined against *Aspergillus niger*, *Memnoniella echinata*, *Chrysosporium merdarium*, *Aspergillus fumigatus*, *Trichoderma viride*, *Rhizopus* spp., *Fusarium nivale*, *Penicillium viridicatum*, *Aspergillus versatile*, and *Aspergillus ferenczii*. The above-mentioned fungi were obtained from the collection of Vytautas Magnus University (Kaunas, Lithuania). All fungi were cultured on yeast extract, peptone, and dextrose medium at 25 °C. The antifungal activity of cereal by-products was tested by an agar well diffusion assay, as described by Bartkiene et al. [10].

### 2.3. Animals and Housing

A 41-day experiment was conducted using 300 (21-day-old) Large White/Norwegian Landrace (LW/NL) piglets (100 piglets in each group) at a ‘Kontvainiai’ farm (Kontvainiai, Lithuania). The experiment started with animals at an initial body weight of 6.9–7.0 kg in control and both treatment groups. The diet of piglets before the trial consisted of crude protein—18.41%, crude fibre—3.24%, crude fat—5.96%, av. lysine—1.46%, av. Methionine—0.60%, av. Tryptophan—0.24%, av. Threonine—0.90%, Ca—0.94%, and total P—0.58%. The weaner piglets’ conditions are described in detail by Vadopalas et al. [7].

### 2.4. Experimental Design and Diets

The piglets were distributed into three groups. Three dietary treatments were compared: (i) a basal diet (C-I—control group), (ii) a basal diet with the addition of extruded–fermented WB (W_ex130/screwspeed25Lpa_) (TG-II), and (iii) a basal diet with sugar beet pulp (TG-III). W_ex130/screw speed 25 Lpa_ and sugar beet pulp were added to the main diet by replacing barley and wheat feed parts. All animal groups were fed wet feed (water and feed ratio of 3/1), and WEDA equipment was used for feeding (Dammann & Westerkamp GmbH, Lutten, Germany).

The piglets’ growth performance was evaluated by testing all 100 piglets in each group; piglets’ blood and faeces’ physico-chemical parameters were evaluated by testing ten piglets from each group; piglets’ faeces’ microbiological parameters were evaluated by testing 40 piglets in each group. The basal feed was formulated according to prescribed nutritional requirements [20]. The feed composition and nutritional value are shown in Table 1. Dietary contents were analysed according to Association of Official Agricultural Chemists AOAC recommendations [21].

### 2.5. Blood Sample Analysis

Piglets’ blood samples were taken according to procedure described by Vadopalas et al. [7]. Blood parameters were evaluated before and after the experiment (on days 28 and 61 of the piglets’ lives) in the accredited laboratory ‘Anteja’ (Klaipeda, Lithuania).

### 2.6. Metagenomics and Microbial Profiling Analysis

Before and after the experiment, faeces from ten piglets from the control and both treatment groups were collected. Library preparation, metagenomic sequencing, and taxonomic characterisation of reads were performed as described previously [7,22]. The results of taxonomic classification were visualised using the interactive online platform https://genome-explorer.com (accessed on 7 February 2022).

### 2.7. Microbiological Analysis of Faecal Samples

The piglets’ faecal samples were collected from 40 animals from each group before (at day 21) and after the experiment (at day 62), stored in vials (+4 °C) with a transport medium (Faecal Enteric Plus, Oxoid, Basingstoke, UK), and analysed on the same day. Evaluation of the microbiological parameters (LAB, total bacteria count (TBC), total enterobacteria count (TEC), and mould and yeast counts (Y/M) was performed according to methods described by Zavistanaviciute et al. [23].

### 2.8. Evaluation of Faecal pH, Dry Matter, and Colour Characteristics

The faecal pH was analysed with a pH metre (Inolab 3, Hanna Instruments, Villafranca Padovana, Italy). The faecal dry matter (DM) was evaluated after drying the samples at 103 ± 2 °C to a constant weight. The colour coordinates were measured at three different points of the faecal sample surface using the CIE L*a*b* system (CromaMeter CR-400, Konica Minolta, Tokyo, Japan).

### 2.9. Analysis of Faecal Volatile Compound Profiles

Faeces for gas chromatography (GC) analysis were prepared by using solid-phase microextraction (SPME). Analysis was performed according to procedure described by Vadopalas et al. [24].

### 2.10. Evaluation of Piglets’ Growth Performance

Group body weight (BW) gain was recorded on days 21, 28, 35, 42, 49, 56 and 62 of age using an electronic weighing system (model type: IT1000, SysTec GmbH, Bergheim, Germany). The feed conversion ratio (FCR) was calculated from feed intake (87% of DM) and BW gain, which was recorded on the same days as BW gain using a WEDA (Dammann & Westerkamp GmbH, Germany) automated feeding system, which has an electronic flowmeter and weighing system. The mortality of piglets during all trials was recorded.

### 2.11. Ethical Statement

All animal procedures were conducted according to the EU Directive of the European Parliament and Council from 22 September 2010 [25] on the protection of animals used for scientific purposes and Requirements for the Keeping, Maintenance, and Use of Animals Intended for Science and Education Purposes, approved by order of the Lithuanian Director of the State Food and Veterinary Service [26]. Research was carried out in accordance with the Republic of Lithuania Act [27].

### 2.12. Statistical Analysis

Data were subjected to multivariate ANOVA using the statistical package SPSS for Windows (Ver.15.0, SPSS, Chicago, IL, USA). Baseline measurements were used as covariates to take the experimental conditions into account. The mean values were compared using Duncan’s multiple range test with a significance level defined at *p* ≤ 0.05. In the tables, the results are presented as mean values with pooled standard errors. Differences in bacterial genera between the groups at the end of experiment were assessed using the Z-test calculator for two population proportions (Social Science Statistics, socscistatistics.com, 2019). Statistical comparisons were considered significant when *p* ≤ 0.05.

## 3. Results and Discussion

### 3.1. Antimicrobial and Antifungal Characteristics of the Cereal By-Products

The antimicrobial properties of extruded and non-extruded WB, non-fermented and fermented with *L. casei* and *L. paracasei* strains, are shown in Table 2. In the comparison of the antimicrobial properties, in all cases, the non-fermented samples were not inhibited by the tested pathogens. The data demonstrate that non-fermented samples had no inhibition on tested micro-organisms except for *S. enterica Infantis*, against which inhibition was detected in W_ConLc_, W_ConLpa_, W_ex115Lc_, and W_ex115Lpa_, with an average diameter of inhibition zones (DIZ) of 10.3 mm.

*Staphylococcus aureus* was inhibited by all the fermented samples. W_ConLc_, W_ConLpa_, W_ex130/screwspeed25Lc_, and W_ex130/screwspeed25Lpa_ inhibited *E. coli.* W_ex115Lc_, W_ex115Lpa_, and W_ex130/screwspeed20Lc_ samples showed inhibition properties against *Bacillus pseudomycoides*. *Aeromonas veronii* was inhibited by most of the fermented samples, except W_ex130/screwspeed20Lpa_. *Cronobacter sakazakii* and *Hafnia alvei* were inhibited by W_ConLc_, W_ConLpa_, W_ex115Lc_, and W_ex115Lpa_ samples, as well as *Enterococcus durans*, inhibited by W_ConLc_, W_ConLpa_, W_ex115Lc_, W_ex115Lpa_, and W_ex130/screwspeed16Lpa_, on average, with the highest DIZ (15.6 mm) being the W_ConLpa_ samples. The strongest antimicrobial properties against *Kluyvera cryocrescens* were shown by W_ex130/screwspeed25Lc_ and W_ex130/screwspeed25Lpa_ samples. *Acinetobacter johnsonii* was inhibited by most of the fermented samples (except W_ex130/screwspeed20Lpa_), with an average DIZ of 26.6 mm. ANOVA indicated that there was a significant effect of the type of LAB applied for the fermentation and extrusion parameters, as well as the interaction of these factors on all the tested pathogenic and opportunistic strains (DIZ; *p* ≤ 0.0001). It was reported that fermented rice bran’s antimicrobial activity against pathogenic and opportunistic strains was due to low pH, organic acidic compounds, and the production of ethanol and bacteriocins [28]. In addition to this, the extrusion of barley, rice, oats, and wheat at 120, 160, and 200 °C and 20% moisture increases the phenolic, hydroxycinnamic, ferulic, and coumaric acids, which show antimicrobial properties [29]. Finally, these tendencies show different mechanisms of antimicrobial activities in the tested samples, as well as different sensitivities for different substances for the different pathogenic strains. However, selected extrusion and fermentation parameters are related to the potential to increase the antimicrobial properties of the cereal by-products, and these changes could be very beneficial when by-products are included in piglets’ nutrition.

When comparing the antifungal properties of WB, in almost all cases, non-fermented samples were unable to inhibit the fungi tested (Table 3).

*Aspergillus niger* and *Trichoderma viride* were not inhibited by any of the WB samples. However, *Memnoniella echinata* had a delay in spore formation, and a small clear zone of inhibition around the punched well (++) was established by W_ConLpa_. A particularly good inhibition of *Chrysosporium merdarium* mycelium growth and sporulation with large clear zones around the punched well was shown by W_ex115Lpa_ (+++). Additionally, a delay of *Chrysosporium merdarium* spore formation with a small clear zone of inhibition around the punched well was caused by W_ex115Lc_, W_ex130/screwspeed16Lc_, and W_ex130/screwspeed16Lpa_ (++). A delay of *Aspergillus fumigatus* spore formation with a small clear zone of inhibition around the punched well was caused by W_ConLc_, W_ConLpa_, W_ex115Lc_, and W_ex115Lpa_ (++). A particularly good inhibition of *Rhizopus* spp. mycelium growth and sporulation with large clear zones around the punched well was caused by W_ConLc_, W_ConLpa_, W_ex115Lpa_, W_ex130/screwspeed25Lc_, and W_ex130/screwspeed25Lpa_ samples (+++). Furthermore, a delay of *Rhizopus* spp. spore formation with a small clear zone of inhibition around the punched well was caused by W_ex130/screwspeed20Lc_ (++). A very good inhibition of *Fusarium nivale* mycelium growth and sporulation was caused by W_ConLc_, W_ex130/screwspeed20Lc_ and W_ex130/screwspeed20Lpa_ (+++). The highest inhibition properties against *Penicillium viridicatum* were shown by W_ex130/screwspeed20Lpa_ (+++). A delay of *Penicillium viridicatum* spore formation with a small clear zone of inhibition around the punched well was caused by W_ConLc_, W_ConLpa_, W_ex115Lc_, W_ex115Lpa_, and W_ex130/screwspeed20Lc_ (++). *Aspergillus versatile* had a delay of spore formation with a small clear zone of inhibition around the punched well caused by W_ConLc_, W_ConLpa_, W_ex115Lc_, W_ex115Lpa_, and W_ex130/screwspeed25Lc_ samples (++). A particularly good inhibition of mycelium growth and sporulation of *Aspergillus ferenczii* was caused by W_ex115_ and W_ex115Lpa_ samples (+++). Finally, W_ConLc_ and W_ex115Lpa_ showed antifungal activities against 7, W_ConLc_ against 6, W_ex130/screwspeed20Lc_ and W_ex130/screwspeed20Lpa_ against 5, W_ex130/screwspeed16Lpa_ against 4, W_ex130/screwspeed16Lc_, and W_ex130/screwspeed25Lc_ and W_ex130/screwspeed25Lpa_ against 3 out of 11 tested fungi. The explanation for these results could be that phenolic compounds of fermented brans inhibit fungal growth by suppressing the biosynthesis of cell wall components, such as glucan, chitin, and mannoproteins, as well as cell membrane components such as ergosterol, as during this process, the cell wall and membrane are destroyed, thus affecting the control of nutrient entry [30,31]. Phenolic compounds of fermented rice bran inhibit the growth of *Penicillium verrucosum*.

Finally, our previous study [5] showed that the lowest mycotoxin concentration and the highest total titratable acidity, as well as the highest viable LAB count, could be found in W_ex130/screwspeed25Lpa_ samples. In addition to the above-mentioned and reported properties, W_ex130/screwspeed25Lpa_ samples showed antimicrobial and antifungal properties (inhibition properties against 5 out of 10 tested pathogenic strains and against 3 out of 11 tested fungi). Valorisation of WB could be performed by using a combination of extrusion and fermentation, leading to safer and higher-functional-value feed material.

### 3.2. Piglets’ Blood Parameters

The blood parameters of the piglets’ are shown in Table 4. In the comparison of immunoglobulin concentrations in piglets’ blood samples at the end of the experiment, significant differences between the group samples in IgA and IgG concentrations were not established. However, a significantly lower IgM concentration in the TG-III group was found in comparison with the C-I and TG-II groups (16.6% lower on average). Higher concentrations of thyroid-stimulating hormone (TSH) in treatment group samples were found in comparison with the control group (19.5 times on average). Additionally, despite the lowest concentration of albumin (ALB) being found on day 21 in the TG-II group piglets’ blood, at the end of the experiment in both treatment groups, a significantly higher ALB concentration was established in comparison with the control group samples (in the TG-II group, 13.1% higher; in the TG-III group, 19.9% higher). Similar tendencies with TP content were found at the end of the experiment, with significantly higher TP concentrations in both treatment group samples (7.3% higher on average in comparison with the control group). In both treatment groups, at the end of the experiment, higher concentrations of alanine aminotransferase (ALT), aspartate aminotransferase (AST), cholesterol (CHOL), high-density lipoprotein cholesterol (HTL), low-density lipoprotein cholesterol (LTL), triglycerides (TGL), glucose (GLU), and triiodothyronine (T3) were found. However, a higher thyroxine (T4) concentration in the control groups was established at the end of the experiment. The lowest concentration of alkaline phosphatase (ALP) was found in TG-II group blood samples at the end of the experiment. However, the highest concentration of ALP was established in TG-III group blood samples, and, in all the cases, a lower ALP concentration at the end of the experiment was found. In the comparison of 21-day-old piglets’ samples, the highest thyroxine (T4) concentration was established in the control and TG-III groups; however, at the end of the experiment, in both treatment groups, the T4 concentration was significantly lower in comparison with the control group.

Feed composition affects the blood parameters of healthy animals [32,33]. Animals’ diets must be balanced in accordance with specific requirements and contain the correct proportions of energy, protein (and specific amino acids), fibre, vitamins, and minerals. In addition to these components, water must be available [34]. However, blood parameters depend on many factors—in addition to diet—including housing, season, age, and breed. [35]. Additionally, different feed components could lead to the formation of different profiles of digesta microbiota, whose composition could be an important factor related to nutrient absorption, as well as blood parameters. Intestinal bacteria play a role in the digestion of dietary fibres and the regulation of the immune response [36]. It was reported that probiotics have beneficial effects on blood parameters and IgG stimulation of weaned piglets’ [37]. According to a few studies, increased plasma immunoglobulin titres indicate stimulation of the humoral mechanism of active specific immunity [38,39]. In our study, the tendency for increased blood IgM levels was found. Higher serum immunoglobulin (Ig) levels may be related to the immune response caused by an infection or, as in our study, activation of Ig production in piglets receiving a diet with fermented WB [40]. Dietary fermented feed supplementation can also play an important role in relieving diarrhoea and producing immune-related effector cells, such as immunoglobulins [41]. The reason that fermented WB can decrease the rate of diarrhoea and change intestinal morphology and intestinal digestive enzyme activity may be due to the fermentation of WB that regulates the composition of the intestinal flora [42]. In this study, feed stock (valorised WB) containing high quantities of viable LAB cells (>6.0 log_10_ CFU/g) was used, whose survival during the transition through the digestive tract was not analysed; however, they could modify microbiota in both viable and inactivated conditions. Furthermore, sugar beet has prebiotic properties possessing compounds (e.g., arabinan, pectic oligosaccharides, etc.) that could be associated with microbiota modification and changes in nutrient absorption. A healthy gut microbiota is associated with beneficial effects on the immune system as well as on thyroid function. In addition, the composition of the gut microbiota is related to the availability of essential micronutrients for the thyroid gland. Iodine, iron, and copper are the most important microelements for thyroid hormone synthesis; selenium and zinc are needed for converting T4 to T3 [43]. Iodothyronine deiodinase enzymes play an important role in the conversion of thyroxine (T4) to its active form, triiodothyronine (T3) [44]. It was reported that deiodinase activity in the intestinal wall could be related to total T3 body levels. An experiment with rats showed the binding of thyroid hormones by gut bacteria, even competing with albumin [45,46]. For this reason, further correlations between piglets’ blood characteristics and faecal microbiological parameters were analysed.

In the present study, piglets fed both supplements had higher blood ALB and TP levels compared with piglets that received basal feed, which suggested an enhancement of the dietary protein bioavailability and improved immune condition of the piglets [47]. In our study, both feed supplements decreased blood creatinine levels and increased the concentration of triglyceride (except in the TG-III group on the 21st day). Triglycerides, along with proteins, may generate low-density lipoprotein cholesterol (LDL) and high-density lipoprotein cholesterol HDL [42]. HDL is able to interfere with the oxidation of LDL and can transfer cholesterol from the macrophage back to the plasma in a process known as reverse cholesterol transport [48]. Creatinine is a marker of renal detoxification function and is closely related to a variety of diseases [49].

### 3.3. Piglets’ Faecal Metagenomics and Microbial Profiling Analysis Results

**Figure 1:**
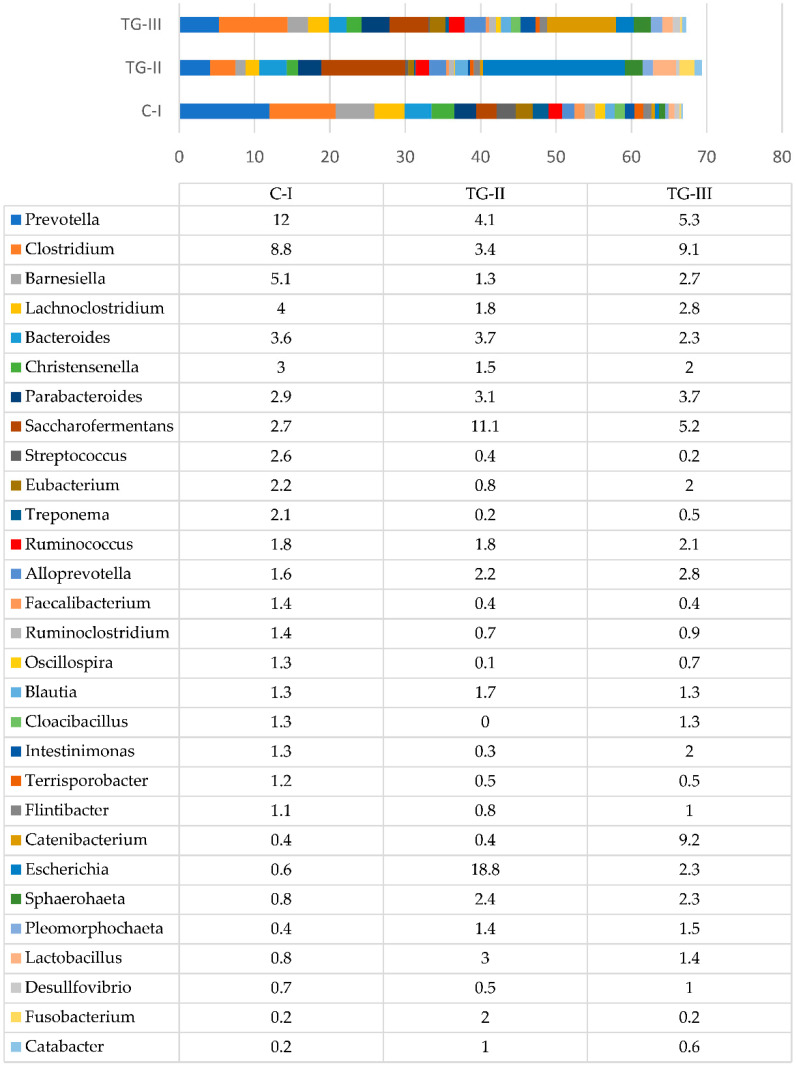
Bacterial genera in faeces of piglets (%) before the experimental treatment (day 21). The data only represent those genera whose numbers from all the bacterial amounts were at least 1% in any of the animal groups.

Microbial profiles of piglets’ faeces before dietary treatment are presented in Figure 1. The most prevalent genera included *Prevotella*, *Clostridium*, *Lachnoclostridium*, *Barnesiella*, *Bacteroides*, *Saccharofermentans*, *Escherichia*, and other normal core microbiota of the gastrointestinal tract of weaned-off pigs.

**Figure 2:**
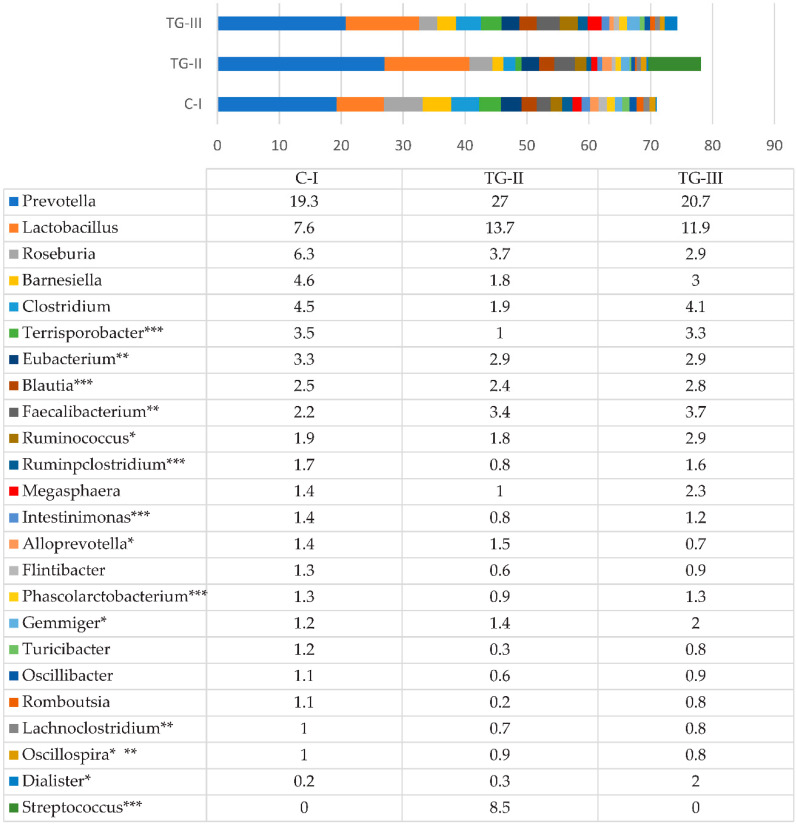
Bacterial composition of piglets’ faeces at the end of the experimental treatment (day 62). The data only represent those genera whose numbers from all the bacterial amounts were at least 1% in any of the animal groups. * Statistically insignificant results between C-I and TG-II. ** Statistically insignificant results between TG-II and TG-III. *** Statistically insignificant results between C-I and TG-III.

Microbial profiles (the most prevalent genera) of the piglets’ faeces at the end of the experimental treatment are presented in Figure 2.

The most prevalent bacterial genus in all of the groups was *Prevotella*, with a prevalence of 19.3–27%. *Lactobacillus* was the second most prevalent genus, and its amount varied from 7.6% in the C-I group to 13.7% in TG-II. Statistically significant differences between all groups, both for *Prevotella* and *Lactobacillus*, were detected, with the highest amount in TG-II and the lowest amount in the C-I group. High amounts (8.5%) of *Streptococcus* were also prevalent in the TG-II group, with the absence of this genus in TG-III and C-I. The most prevalent *Streptococcus* species included *S. gallolyticus*, *S. lutetiensis*, *S. alactolyticus*, and *S. infantarius*. The other core bacteria characteristic of piglets’ gastrointestinal tracts, including *Barnesiella*, *Clostridium*, *Faecalibacterium*, *Ruminococcus*, were detected in different numbers. Of those bacteria, the most prevalent genera in the TC-II group were *Roseburia* and (3.7%) and *Faecalibacterium* (3.4%); in the TC-III group, *Clostridium* (4.1%) and *Faecalibacterium* (3.7%) were most prevalent; whereas, among the control group, *Roseburia* (6.3%), *Barnesiella* (4.6%), and *Clostridium* (4.5%) were most prevalent.

As a principal genus of the gut microbiota, *Prevotella* spp. are major contributors to host physiology. Many correlative studies have associated members of the genus *Prevotella* with positive outcomes in pig production, including growth performance and immune response [50,51]. There is evidence correlating an increase in intestinal *Prevotella* and enhanced vaccine response in pigs. For instance, a positive correlation was detected between the abundance of *Prevotella* and vaccine responsiveness against *Mycoplasma hyopneumoniae* [52]. In the present study, *Prevotella copri* was the most prevalent species in all animal groups on day 62, with amounts ranging from 9.1% (C-I group) to 18.7% (TG-II group) of all bacterial species detected in pig faeces. Previous data indicated that a higher number of this bacterial species was associated with greater weight gain [53]. Recent investigations demonstrated that *P. copri* has close interactions with other microbial community members of the gut in both pre- and post-weaning pigs [54]. Due to the importance of this genus to pigs’ intestinal health, researchers have discussed the possibility of developing more effective probiotics from *Prevotella* spp. [50]. This study demonstrates that extruded–fermented WB as well as sugar beet processing by-products as a feed supplement significantly increase the numbers of *Prevotella* in the body without the necessity of adding probiotic strains and/or prebiotics.

The other genus that plays an important role as a natural probiotic bacterium is *Lactobacillus*, which was more prevalent in the treated animal groups (*p* < 0.05) compared to the control group based on the results of metagenomic sequencing. *Lactobacillus* are known for the possibility of reducing the number of pathogenic bacteria in the gut and even providing a potential alternative to antibiotic strategies [51]. A significant increase of *Lactobacillus* because of the feed supplemented by fermented by-products can increase intestinal immunity in pigs without the necessity of adding probiotics or other supplements, therefore having a positive economic effect. *Streptococcus* is the other genus that depends on the core of the normal microbiota of pigs [55]. *Streptococcus* spp. are also used as probiotic bacteria [56,57]. This genus, however, was only detected in high amounts in the faeces of TG-II group pigs, demonstrating different microbial community variations when animal feed was supplemented by different fermented by-products. Overall, almost half (49.2%) of the microbial composition in the TG-II group consisted of *Prevotella*, *Lactobacillus*, and *Streptococcus*—core micro-organisms that are known for their probiotic properties and ability to regulate the micro-organism community in the gut of animals. Microbiota in both treatment groups also contained higher numbers of *Faecalibacterium*, which is reported as a promising probiotic bacteria [57] and is prevalent in healthy pigs [58]. Higher amounts of *Roseburia*, *Barnesiella*, and *Clostridium* bacteria were detected in the control group in comparison with the treatment groups. Although these bacteria are known as normal core microbiota in pigs, their significance is still being investigated, whereas the opinions about their effect are controversial and require more studies.

Considering microbial profiles in tested animals, it may be stated that appropriately selected processed by-products may have a significant influence on the microbial composition of healthy animals. These products can be treated as probiotics, symbiotics (and/or extruded–fermented WB), and prebiotics (sugar beet pulp), as they increase the numbers of probiotic bacterial species, which are also known as an indicator of good immune status.

### 3.4. Microbiological Parameters of Piglet Faecal Samples

The microbiological parameters of the piglets’ faecal samples are shown in Table 5. In the comparison of the total count of enterobacteria (TCE) in piglets’ faeces on day 21, the lowest TCE number was found in the TG-II group (9.0% lower on average in comparison with the control and TG-III groups). However, at the end of the experiment, a significantly lower TCE count in both treatment groups was established in comparison with the control (20.5% lower in TG-II; 12.9% lower in TG-III).

In comparison, the LAB count in piglets’ faeces before and after the experiment, in all groups, was higher at the end of the experiment. In all three groups, at the end of the experiment, a higher LAB count in the TG-II and TG-III groups was found in comparison with the control group (13.7% higher on average in comparison to the control group).

Despite the day and treatment interaction not having a significant impact on the faecal microbiological parameters analysed, strong correlations between some faecal microbiological parameters and blood parameters were found. Strong positive correlations between the IgM concentration in piglets’ blood and LAB, as well as the *Enterococcus faecalis* count in faecal samples, were established (r = 0.764, *p* < 0.0001 and r = 0.764, *p* < 0.0001, respectively). Additionally, a strong positive correlation between blood ALB and mould/yeast number in faeces was found (r = 0.731, *p* < 0.001).

The micro-organism profile in the intestine is related to the environment and feed nutrients in the intestinal lumen [59], as well as the animal’s health status and growth performance [60]. It was reported that the addition of probiotics, as well as prebiotics to feed, could modify the intestinal microbiota and improve the immune function in piglets [61,62,63,64]. Our study showed that the feed composition could be successfully modified by including food industry by-products, which possess antimicrobial, antifungal (extruded and fermented WB), and/or prebiotic (sugar beet pulp) properties. Feed additives (probiotics, prebiotics, and/or symbiotics) are associated with effective modulation of the intestinal microbiota in piglets [62,65]. Additionally, it was reported that probiotics decrease *E. coli* and improve the immune response of piglets [66]. Our results agree with these findings, as a strong positive correlation between IgM concentration in piglets’ blood and LAB count in piglets’ faeces was established. Finally, local food industry by-products, which possess probiotic and/or prebiotic properties, could lead to desirable faecal microbial population changes without probiotic and/or prebiotic addition to piglets’ diet.

### 3.5. Faecal pH, Dry Matter, and Colour Coordinates

The physico-chemical parameters (pH, DM, and colour coordinates) of the piglets’ faeces are shown in Table 6**.** Despite there being no significant differences at the beginning of the experiment in piglets’ faecal pH, at the end of the experiment, the lowest pH was found in the TG-III group samples (5.83). In the comparison of faecal DM, at the beginning of the experiment, the control group’s faecal DM was the lowest; however, at the end of the experiment, the lowest DM was found in the TG-II group samples (13.6% lower than the control group, and 16.4% lower than the TG-III group). The highest lightness (L*), redness (a*), and yellowness (b*), at the end of the experiment, occurred in the control group faeces. Additionally, strong positive correlations were found (r = 0.686, *p* = 0.002; r = 0.646, *p* = 0.004; r = 0.653, *p* = 0.003, respectively) between faecal pH and microbiological parameters (TCE, TCM, M/Y count). In addition, strong positive correlations were established between faecal pH and DM and between faecal DM and texture hardness (r = 0.643, *p* = 0.004 and r = 0.718, *p* = 0.001, respectively).

The inclusion of different dietary fibre sources in feed is a promising strategy to stabilise the gut health of weaner piglets [67]. Different dietary fibre compounds influence digestion and fermentation processes [68,69,70,71], as well as lead to differences in pH, DM, and colour coordinates of the faeces. It was reported that the characteristics of dietary fibre are an important factor that has an influence on piglets’ faecal DM content [67]. Soluble dietary fibre (typical for sugar beet pulp) can reduce water absorption and decrease nutrient digestibility [72]. A high-viscosity diet is related to increased digesta viscosity [73], and the latter could be related to the higher concentration of soluble non-starch polysaccharides in feeds’ dietary fibre [74,75]. Additionally, faecal texture parameters could be related to the activity of digestive enzymes [76]. In relation to changes in pH and enzyme activity, colour coordinates could differ. This could be associated with the colour compounds’ degradation and/or new complexes’ formation. In this study, faecal lightness (L*) showed strong positive correlations with TCE, TCM, *Enterococcus faecalis*, and M/Y count in faeces, as well as the pH of the faecal samples (r = 0.723, *p* = 0.001; r = 0.715, *p* = 0.001; r = 0.717, *p* = 0.001; r = 0.622, *p* = 0.006; r = 0.851, *p* < 0.0001, respectively). However, between the other faecal colour coordinates (a* and b*) and microbiological as well as physico-chemical parameters, no correlations were established.

Finally, faecal consistency was within a physiological range throughout the feeding experiment period, and the dietary impact on faecal consistency was not accompanied by any negative effects on animal health.

### 3.6. Piglets’ Faecal Volatile Compound Profile

Piglets’ faecal volatile compounds (% from the total volatile compounds) whose content in profile was higher than 1.0% are shown in Table 7, and the whole volatile compound profile of the piglets’ faeces is given in Supplementary File S2. Table 1. Butanoic acid, 3-methyl-; butyric acid (2-methyl-) and isothiocyanate (3-butenyl-) were found just in the treatment group (TG-II and TG-III) samples after 62 days. Pentanoic acid and benzene, 1,3-bis(1,1-dimethylethyl)- were indicated in most of the samples, except in the control group at the beginning of the experiment. *p*-Cresol, indole, 1H-indole, and 3-methyl- were predominant volatile compounds in all the faecal samples. Additionally, butylated hydroxytoluene and n-nonadecanol-1 were found in all groups’ faeces. Some compounds were specific to certain groups: butanoic acid and 2-methyl- were found in the C-I group’s faeces after 62 days (8.07%), phosphonic acid (p-hydroxyphenyl)- was found in the TG-III group after 21 days (3.33%), and phenol was found in the control group faeces at the beginning of the experiment (9.40%).

Correlations between the separate piglet faecal volatile compounds and microbiological parameters are shown in Table 8. Strong positive correlations were found between the LAB count in piglets’ faeces and butanoic acid; butanoic acid, 3-methyl-; butyric acid (2-methyl-); pentanoic acid were found, as well as a strong negative correlation between the LAB count and n-nonadecanol-1. Contrary to these findings, correlations between volatile compounds and TCM were not established. A moderate negative correlation between TCE in faeces and isothiocyanate <3-butenyl-> was found, as well as moderate positive correlations between the *Enterococcus faecalis* count in piglets’ faeces and butanoic acid; pentanoic acid; hexanoic acid and 1H-indole, 3-methyl- being established.

Faeces is the end-product of nutrient conversion, which includes digestive and excretory processes as well as microbial metabolism [18]. Separate volatile compounds (VC) formation in faeces could be related to changes in microbial metabolism [77]. It was reported that the characterisation of the faecal VC profile might lead to the development of a rapid, non-invasive tool to monitor gastrointestinal functionality [78]. Pérez-Calvo hypothesised that VC formation could be associated with the microbial profile [18]. Our study showed that some correlations between separate VCs and micro-organism numbers in faeces were significant. Finally, very promising results were obtained; however, further studies are needed to indicate separate micro-organisms’ strains’ relation to the separate VCs.

### 3.7. Piglets’ Growth Performance

**Figure 3:**
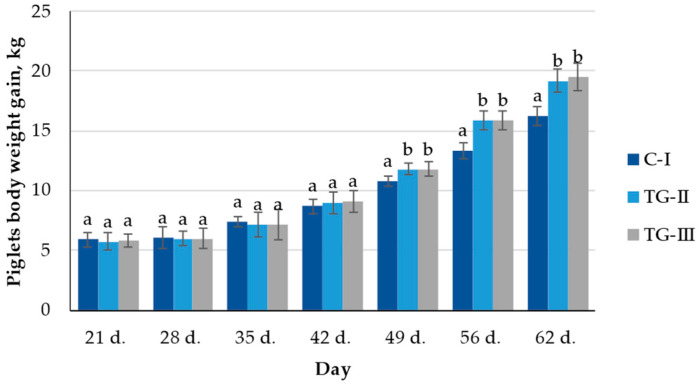
Piglets’ body weight (BW) gain (kg) (^a,b^ different letters indicate differences among treatments, *p* < 0.05; C-I—control group TG-II—treatment group with extruded–fermented wheat bran addition; TG-III—treatment group with sugar beet pulp).

In the comparison of piglets’ body weight (BW) gain, no significant differences between the groups after 21, 28, and 35 days were established (Figure 3). However, after 42 days, a significantly higher BW occurred in the TG-III group piglets (2.8% higher on average in comparison with the control and TG-II groups). After 49, 56, and 62 days, both treatment groups showed a significantly higher BW in comparison with the control group (9.1, 16.4, and 15.9% on average, respectively).

**Figure 4:**
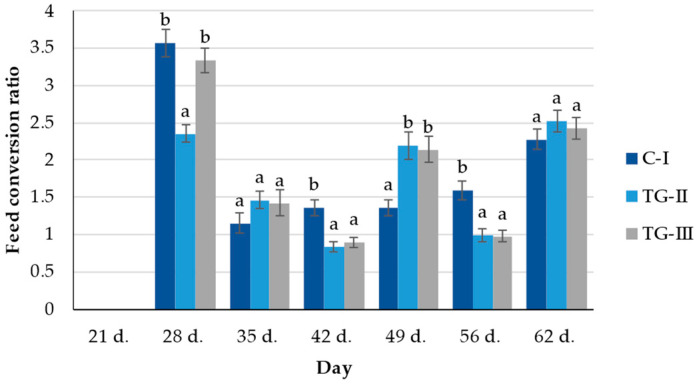
The feed conversion ratio (FCR) (^a,b^ different letters indicate differences among treatments, *p* < 0.05; C-I—control group TG-II—treatment group with extruded–fermented wheat bran addition; TG-III—treatment group with sugar beet pulp).

In the comparison of feed conversion ratio (FCR), after 28 days, the lowest FCR was found in the TG-II group; however, after 35 days, no significant differences between the groups were established (Figure 4). After 42 days, the control group showed the highest FCR; however, after 49 days, opposite tendencies were found, and the highest FCR was found in both treatment groups. After 56 days, the control group showed a significantly higher FCR; however, after 62 days, no significant differences between the groups’ FCR were established (2.40 on average).

**Figure 5:**
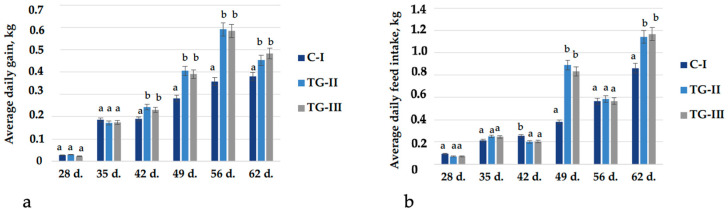
(**a**) The average daily gain (ADG, kg), (**b**) the average daily feed intake (ADFI, kg) (^a,b^ different letters indicate differences among treatments, *p* < 0.05; C-I—control group TG-II—treatment group with extruded–fermented wheat bran addition; TG-III—treatment group with sugar beet pulp; d.—day).

In the comparison of the average daily gain (ADG, kg), after 28 and 35 days, significant differences between the groups’ ADG were not found; however, after 42, 49, 56, and 62 days, a significant higher ADG of both treatment groups was found in comparison with the control group (Figure 5a). In the comparison of the average daily feed intake (ADFI, kg), after 28 and 35 days, significant differences were not found; however, after 42 days, a lower ADFI of both treatment groups was found (Figure 5b). Opposite tendencies after 49 and 62 days were established, and in both treatment groups, a significant higher ADFI was found.

**Figure 6:**
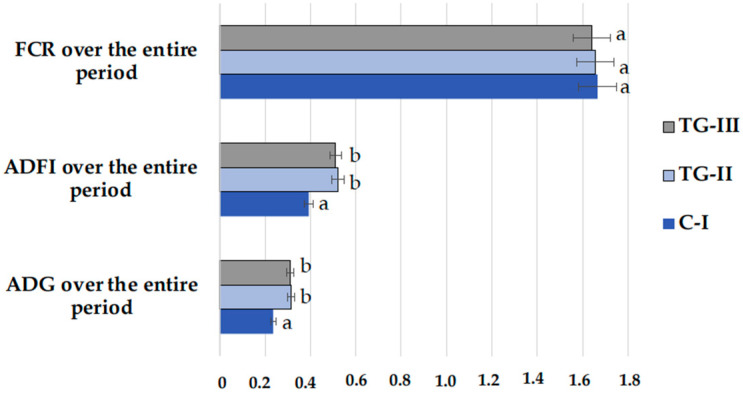
Feed conversion ratio (FCR), average daily feed intake (ADFI), and average daily gain (ADG) over the entire period (^a,b^ different letters indicate differences among treatments, *p* < 0.05; C-I—control group TG-II—treatment group with extruded–fermented wheat bran addition; TG-III—treatment group with sugar beet pulp).

In the comparison of the FCR, ADFI, and ADG over the entire period, significant higher ADFI and ADG in both treatment groups were found in comparison with the control group (Figure 6). However, between the groups, significant FCR differences were not established. The conversion of feed into weight gain is very important in pig production. How well this is achieved is described by the FCR value. However, FCR is a biological factor that can be influenced by the feeding practice, environmental control, genetics, health status, etc. In addition, high-value and (or) better functionality feed or feed additives used for controlling gut health status can achieve the same or higher FCR.

The composition of the compound feed did not have an influence on piglet survival. The piglet mortality rate in the C-I group was 1.6%, 3.3% in the TG-II group, and 1.3% in the TG-III group. The mortality of piglets was not associated with gastrointestinal pathologies.

Despite differences between the diets, a balanced nutrient content regarding energy and nutrient requirements was suggested. However, the significantly higher BW of the treatment groups could be associated with desirable changes in the micro-organism populations in the piglets’ faeces, which led to better nutrient absorption and higher BW.

## 4. Conclusions

An extrusion and fermentation combination is a suitable strategy for WB valorisation, as pre-treated WB showed desirable antimicrobial and antifungal properties. Both the tested treatments showed a positive influence on piglets’ faecal microbiological parameters (reduced total enterobacteria and increased LAB count). Additionally, strong positive correlations were found between the LAB count in piglets’ faeces and the separate VCs (butanoic acid; butanoic acid, 3-methyl-; butyric acid (2-methyl-); pentanoic acid). Further studies are needed to indicate which VC could be directly associated with the piglets’ health status. Despite differences between the diets, a balanced nutrient content regarding energy and nutrient requirements is suggested; however, the significantly higher ADG of the treatment groups was associated with desirable changes in micro-organism populations in the piglets’ faeces. Further research is needed to identify which compounds incorporated into the piglets’ feed showed the main desirable properties. Finally, the tested local feed material could be used to enhance piglet nutrition and lead to more sustainable livestock production.

## Figures and Tables

**Table 1:** Diet composition.

Ingredients (%)	C-I	TG-II	TG-III
Barley	46.78	45.22	44.48
Wheat	25.70	24.10	24.10
Pea	3.00		
W_ex130/screwspeed25Lpa_	-	3.00	-
Sugar beet pulp (dried)	-	-	3.00
Soybean meal	4.20	4.20	4.70
Potato protein	3.50		
Soybean protein concentrate	3.00		
Whey powder	6.00		
Sunflower oil	3.12	3.22	3.44
Limestone	1.43		
NaCl	0.34		
Monocalcium phosphate	0.14		
Lysine sulphate, L-Lysine 54.6%	1.36	1.39	1.39
DL-Methionine, 99%	0.38	0.41	0.43
Choline chloride 75%, liquid	0.05		
^1^ Vitamins and trace elements (premix)	1.00		
Nutritional value			
ME swine (MJ/kg)	13.88		
Crude protein (%)	18.42	18.41	18.39
Crude fat (%)	5.96		
Crude fibre (%)	3.00	3.24	3.47
NDF	12.58	12.66	12.84
ADF	3.82	3.98	4.09
Lysine (%)	1.57		
Methionine (%)	0.65		
Threonine (%)	0.99	1.00	1.00
Tryptophan (%)	0.29		
Methionine + Cystine (%)	0.90		
Ca (%)	0.94		
Total P (%)	0.58	0.57	0.58
Available P (%)	0.37	0.36	0.36
Na (%)	0.17	0.18	0.18

(i) A basal diet (C-I—control group); (ii) a basal diet with extruded–fermented wheat bran addition (TG-II); (iii) a basal diet—sugar beet pulps, dried (TG-III); W_ex130/screwspeed25Lpa_-extruded at 130 °C and 25 rpm and fermented with *Lactobacillus paracasei* wheat bran. ^1^ Composition of premix per 1 kg of feed: vitamin A—18.180 IU; vitamin D3—2.000 IU; vitamin E—160 mg/kg; vitamin K3—5.00 mg; thiamine—3.64 mg; riboflavin—9.16 mg; choline chloride—4 mg; pyridoxine—4.60 mg; vitamin B12—0.05 mg; niacin—40.54 mg; pantothenic acid—22.54 mg; folic acid—1.80 mg; biotin—0.2 mg; Fe—150 mg; Cu—101 mg; Zn—100 mg; Mn—84 mg; I—0.72 mg; Co—0.50 mg; Se—0.40 mg. For all groups, the following were added to the compound feed: NSP Enzyme-Rovabio Excel AP-50 g/t, endo-1,4-β-xylanase 1100 VU/kg of feed, endo-1,3 (4)-glucanase, 1500 VU/kg of feed and Phytase Axtra PHY 10,000 TPT 2—130 g/t-1300 FTU/kg feed.

**Table 2:** Antimicrobial properties of extruded and non-treated wheat processing by-products, non-fermented and fermented with *L. casei* and *L. paracasei* strains.

Cereal By-Product Samples	Opportunistic and Pathogenic Strains									
	1	2	3	4	5	6	7	8	9	10
	Diameter of Inhibition Zones, mm									
W_Con_	nd	nd	nd	nd	Nd	nd	nd	nd	nd	nd
W_ConLc_	10.2 ± 0.5 ^a^	14.5 ± 0.1 ^d^	9.2 ± 0.2 ^a^	nd	25.9 ± 0.6 ^d^	10.2 ± 0.3 ^a^	28.3 ± 0.3 ^c^	14.5 ± 0.6 ^c^	16.4 ± 0.5 ^c^	29.4 ± 0.3 ^d^
W_ConLpa_	9.3 ± 0.4 ^a^	13.0 ± 0.2 ^c^	9.2 ± 0.3 ^a^	nd	30.8 ± 0.7 ^e^	10.3 ± 0.2 ^a^	40.4 ± 0.3 ^d^	15.6 ± 0.5 ^c^	16.5 ± 0.4 ^c^	31.6 ± 0.9 ^e^
W_ex115_	nd	nd	nd	nd	nd	nd	nd	nd	nd	nd
W_ex115Lc_	10.6 ± 0.1 ^a^	12.2 ± 0.3 ^b^	nd	14.1 ± 0.2 ^c^	13.6 ± 0.6 ^b^	10.4 ± 0.3 ^a^	12.2 ± 0.3 ^b^	11.0 ± 0.3 ^b^	14.9 ± 0.5 ^b^	27.5 ± 0.5 ^c^
W_ex115Lpa_	11.1 ± 0.3 ^b^	12.3 ± 0.3 ^b^	nd	13.3 ± 0.2 ^b^	13.0 ± 0.3 ^b^	10.6 ± 0.2 ^a^	11.1 ± 0.4 ^a^	11.3 ± 0.2 ^b^	14.4 ± 0.4 ^b^	25.9 ± 0.9 ^c^
W_ex130/screwspeed16_	nd	nd	nd	nd	nd	nd	nd	nd	nd	nd
W_ex130/screwspeed16Lc_	nd	10.3 ± 0.2 ^a^	nd	nd	12.3 ± 0.6 ^b^	nd	nd	nd	11.2 ± 0.3 ^a^	26.6 ± 0.7 ^c^
W_ex130/screwspeed16Lpa_	nd	13.1 ± 0.4 ^c^	nd	nd	15.6 ± 0.5 ^c^	nd	nd	9.6 ± 0.3 ^a^	14.1 ± 0.2 ^b^	27.6 ± 0.8 ^c^
W_ex130/screwspeed20_	nd	nd	nd	nd	nd	nd	nd	nd	nd	nd
W_ex130/screwspeed20Lc_	nd	13.3 ± 0.3 ^c^	nd	10.2 ± 0.3 ^a^	11.0 ± 0.4 ^a^	nd	nd	nd	nd	20.9 ± 0.3 ^a^
W_ex130/screwspeed20Lpa_	nd	nd	nd	nd	nd	nd	nd	nd	nd	nd
W_ex130/screwspeed25_	nd	nd	nd	nd	nd	nd	nd	nd	nd	nd
W_ex130/screwspeed25Lc_	nd	14.2 ± 0.3 ^d^	15.3 ± 0.5 ^b^	nd	10.6 ± 0.3 ^a^	nd	nd	nd	30.5 ± 0.3 ^e^	23.3 ± 0.3 ^b^
W_ex130/screwspeed25Lpa_	nd	17.3 ± 0.4 ^e^	15.3 ± 0.6 ^b^	nd	13.2 ± 0.4 ^b^	nd	nd	nd	20.6 ± 0.5 ^d^	26.7 ± 0.6 ^c^

W—wheat bran; Con—non-extruded wheat bran; Lc—fermented with *Lactobacillus casei*; Lpa—fermented with *Lactobacillus paracasei*; ex115—extruded at 115 °C, screw speed 16 rpm; ex130/screwspeed16—extruded at 130 °C, screw speed 16 rpm; ex130/screwspeed20—extruded at 130 °C, screw speed 20 rpm; ex130/screwspeed25—extruded at 130 °C, screw speed 25 rpm; 1. *Salmonella enterica Infantis* LT 101; 2. *Staphylococcus aureus* LT 102; 3. *E. coli* LT 103; 4. *Bacillus pseudomycoides* LT 104, 5. *Aeromonas veronii* LT 105; 6. *Cronobacter sakazakii* LT 106; 7. *Hafnia alvei* LT 107; 8. *Enterococcus durans* LT 108; 9. *Kluyvera cryocrescens* LT 109; 10. *Acinetobacter johnsonii* LT 110; nd—not determined. Data are presented as means (*n* = 5) ± SE. ^a–e^—mean values within a row denoted with different letters are significantly different (*p* ≤ 0.05).

**Table 3:** Antifungal properties of extruded and non-treated wheat processing by-products, non-fermented and fermented with *L. casei* and *L. paracasei* strains.

Cereal by-Product Samples	Fungi										
	1	2	3	4	5	6	7	8	9	10	Total Fungi Inhibited
	Inhibition of Fungi by the Wheat Bran Samples										
W_Con_	-	-	-	-	-	-	-	-	-	-	0
W_ConLc_	-	+	-	++	-	+++	+++	++	++	-	7
W_ConLpa_	-	++	-	++	-	+++	++	++	++	-	6
W_ex115_	-	-	-	-	-	-	-	-	-	-	0
W_ex115Lc_	-	-	-	++	-	-	+	++	++	-	4
W_ex115Lpa_	-	-	+++	++	-	+++	+	++	++	+++	7
W_ex130/screwspeed16_	-	-	-	-	-	-	-	-	-	-	0
W_ex130/screwspeed16Lc_	-	+	++	-	-	-	-	-	+	-	3
W_ex130/screwspeed16Lpa_	-	+	++	-	-	+	-	-	+	-	4
W_ex130/screwspeed20_	-	-	-	-	-	-	-	-	-	-	0
W_ex130/screwspeed20Lc_	-	-	+	-	-	++	+++	++	+	-	5
W_ex130/screwspeed20Lpa_	-	-	+	-	-	+	+++	+++	+	-	5
W_ex130/screwspeed25_	-	-	-	-	-	-	-	-	-	-	0
W_ex130/screwspeed25Lc_	-	-	-	-	-	+++	-	+	++	-	3
W_ex130/screwspeed25Lpa_	-	-	-	-	-	+++	-	+	+	-	3

W—wheat bran; Con—non-extruded wheat bran; Lc—fermented with *Lactobacillus casei*; Lpa—fermented with *Lactobacillus paracasei*; ex115—extruded at 115 °C, screw speed 16 rpm; ex130/screwspeed16—extruded at 130 °C, screw speed 16 rpm; ex130/screwspeed20—extruded at 130 °C, screw speed 20 rpm; ex130/screwspeed25—extruded at 130 °C, screw speed 25 rpm; 1. *Aspergillus niger*; 2. *Memnoniella echinata*; 3. *Chrysosporium merdarium*; 4. *Aspergillus fumigatus*; 5. *Trichoderma viride*; 6. *Rhizopus*; 7. *Fusarium nivale*; 8. *Penicillium viridicatum*; 9. *Aspergillus versatile*; 10. *Aspergillus ferenczii*. Interpretation of inhibition of fungi by the wheat bran samples: (-) no inhibition, (+) delay of spore formation, (++) delay of spore formation with a small clear zone of inhibition around the punched well, (+++) a very good inhibition of mycelium growth and sporulation with large clear zones around the punched well.

**Table 4:** Blood parameters of the piglets.

Blood Parameters	Day	C-I	TG-II	TG-III	*p* Day × Treatment Interaction
IgA	21	0.330 ^Aa^	0.330 ^Aa^	0.330 ^Aa^	1
	62	0.330 ^Aa^	0.330 ^Aa^	0.330 ^Aa^	
IgM	21	0.240 ^Ab^	0.272 ^Ac^	0.230 ^Aa^	0.771
	62	0.442 ^Bb^	0.460 ^Bb^	0.376 ^Ba^	
IgG	21	3.13 ^Ba^	3.09 ^Ba^	2.95 ^Ba^	0.961
	62	1.93 ^Aa^	1.70 ^Aa^	1.79 ^Aa^	
TSH	21	0.011 ^Ba^	0.013 ^Aa^	0.013 ^Aa^	0.002
	62	0.001 ^Aa^	0.020 ^Bb^	0.019 ^Bb^	
ALB	21	37.6 ^Bb^	34.8 ^Aa^	36.4 ^Ab^	0.019
	62	30.6 ^Aa^	35.2 ^Ab^	38.2 ^Ac^	
TP	21	49.0 ^Bc^	45.4 ^Aa^	47.6 ^Ab^	0.115
	62	45.8 ^Aa^	49.0 ^Bb^	49.8 ^Bb^	
UREA	21	3.83 ^Ba^	3.78 ^Ba^	2.06 ^Aa^	0.208
	62	2.00 ^Aa^	1.80 ^Aa^	1.98 ^Aa^	
CREA	21	92.2 ^Bc^	86.6 ^Ba^	88.1 ^Bb^	0.889
	62	54.1 ^Aa^	43.5 ^Aa^	52.5 ^Aa^	
ALT	21	35.6 ^Aa^	48.2 ^Ab^	50.6 ^Ab^	0.727
	62	47.0 ^Ba^	68.0 ^Bb^	70.0 ^Bb^	
AST	21	30.8 ^Aa^	38.0 ^Ab^	42.0 ^Ab^	0.655
	62	26.2 ^Aa^	41.6 ^Ab^	38.0 ^Ab^	
ALP	21	323.0 ^Ba^	407.0 ^Bb^	462.0 ^Bc^	0.189
	62	252.0 ^Ab^	225.0 ^Aa^	310.0 ^Ac^	
TBI	21	12.5 ^Ba^	22.2 ^Ab^	9.40 ^Ba^	0.160
	62	3.00 ^Aa^	3.00 ^Aa^	6.68 ^Aa^	
CHOL	21	3.52 ^Ba^	5.45 ^Bb^	2.93 ^Aa^	0.099
	62	2.07 ^Aa^	2.48 ^Ab^	2.69 ^Ab^	
HTL	21	0.878 ^Ba^	0.948 ^Aa^	1.04 ^Aa^	0.913
	62	0.800 ^Aa^	0.950 ^Ab^	0.992 ^Ab^	
LTL	21	2.19 ^Bb^	3.13 ^Bc^	1.42 ^Aa^	0.122
	62	1.05 ^Aa^	1.25 ^Ab^	1.32 ^Ab^	
TGL	21	0.864 ^Ba^	1.78 ^Bb^	0.786 ^Aa^	0.047
	62	0.502 ^Aa^	0.680 ^Ab^	0.836 ^Ac^	
GLU	21	5.66 ^Ab^	4.52 ^Aa^	5.70 ^Ab^	0.129
	62	5.94 ^Ba^	6.58 ^Bb^	7.00 ^Bb^	
T3	21	0.803 ^Ac^	0.424 ^Aa^	0.662 ^Ab^	0.077
	62	1.02 ^Ba^	1.52 ^Bb^	1.65 ^Bb^	
T4	21	3.53 ^Ab^	2.45 ^Aa^	3.29 ^Ab^	0.132
	62	3.28 ^Ab^	3.00 ^Aa^	3.08 ^Aa^	

(i) a basal diet (C-I—control group); (ii) a basal diet with extruded–fermented wheat bran addition (TG-II); (iii) a basal diet with dried sugar beet pulp (TG-III); IgA, IgM, IgG—immunoglobulin, g/L; TSH—thyroid-stimulating hormone; ALB—albumin, g/L; TP—total protein, g/L; UREA—urea, mmol/L; CREA—creatinine, µmol/L; ALT—alanine aminotransferase, U/L; AST—aspartate aminotransferase, U/L; ALP—alkaline phosphatase, U/L; TBI—total bilirubin, pmol/L; CHOL—cholesterol, mmol/L; HTL—high-density lipoprotein cholesterol, mmol/L; LTL—low-density lipoprotein cholesterol, mmol/L; TGL—triglycerides, mmol/L; GLU—glucose, nmol/L; T3—triiodothyronine, nmol/L; T4—thyroxine, µ d/L. ^A,B^ different capitals indicate significant time-related differences (*p* < 0.05); ^a,b,c^ different letters indicate differences among treatments (*p* < 0.05) Data are presented as means (*n* = 10/group). Baseline measurements were completed on day 21, before the start of the feeding experiment.

**Table 5:** Microbiological parameters in the samples of pig faeces.

Micro-Organism Count, log_10_ CFU/g	Day	C-I	TG-II	TG-III	*p* Day × Treatment Interaction
TCE	21	7.41 ^Ab^	6.84 ^Ba^	7.62 ^Bb^	0.301
	62	7.69 ^Ac^	6.11 ^Aa^	6.70 ^Ab^	
LAB	21	5.11 ^Aa^	5.91 ^Ab^	5.28 ^Aa^	0.704
	62	5.95 ^Aa^	6.96 ^Bb^	6.83 ^Bb^	
TCM	21	7.77 ^Aa^	7.50 ^Aa^	8.22 ^Aa^	0.471
	62	8.32 ^Ba^	7.30 ^Aa^	7.94 ^Aa^	
*Enterococcus faecalis*	21	3.91 ^Aa^	4.26 ^Aa^	4.34 ^Aa^	0.550
	62	5.34 ^Ba^	4.53 ^Aa^	5.26 ^Aa^	
Y/F	21	5.06 ^Aa^	4.1 ^Aa^	5.42 ^Aa^	0.707
	62	4.53 ^Aa^	4.54 ^Aa^	5.07 ^Aa^	

(i) A basal diet (C-I—control group); (ii) a basal diet with extruded–fermented wheat bran addition (TG-II); (iii) a basal diet with dried sugar beet pulp (TG-III). The data are presented by the means (*n* = 40/group). 21 d—the beginning of the experiment; 62 d—the end of experiment. CFU, colony-forming units; LAB, lactic acid bacteria count; TCE, total count of enterobacteria; TCM, total count of aerobic and facultative anaerobic micro-organisms; Y/F, yeast/fungi. ^A,B^ Different capitals indicate significant time-related differences (*p* < 0.05). ^a,b,c^ Different letters indicate differences among treatments (*p* < 0.05).

**Table 6:** The pH, dry matter, and colour coordinates of the piglets’ faeces.

	Day	C-I	TG-II	TG-III	*p* Day × Treatment Interaction
pH	21	6.61 ^Ba^	6.98 ^Ba^	7.02 ^Ba^	0.742
	62	6.24 ^Ab^	6.22 ^Ab^	5.83 ^Aa^	
Dry matter (%)	21	21.52 ^Aa^	24.72 ^Bb^	22.83 ^Bb^	<0.001
	62	19.62 ^Ab^	16.96 ^Aa^	20.29 ^Ac^	
Texture (mJ)	21	0.200 ^Bb^	0.200 ^Bb^	0.180 ^Ba^	0.790
	62	0.100 ^Aa^	0.100 ^Aa^	0.100 ^Aa^	
**Colour Coordinates, NBS:**					
L*	21	34.62 ^Aa^	35.99 ^Ba^	34.20 ^Ba^	0.598
	62	37.81 ^Bc^	34.34 ^Ab^	31.71 ^Aa^	
a*	21	0.200 ^Ab^	0.800 ^Bc^	0.010 ^Aa^	<0.001
	62	4.28 ^Bc^	0.170 ^Aa^	0.460 ^Bb^	
b*	21	9.20 ^Ab^	6.78 ^Aa^	6.88 ^Aa^	0.004
	62	17.2 ^Bb^	7.20 ^Ba^	7.61 ^Ba^	

(i) A basal diet (C-I—control group); (ii) a basal diet with extruded–fermented wheat bran addition (TG-II—treatment group II); (iii) a basal diet—sugar beet pulp, dried (TG-III—treatment group III). The data are presented by the means (*n* = 40/group). Next, 21 day—the beginning of the experiment; 62 d—the end of experiment. L*, lightness; a*, redness or −a*, greenness; b*, yellowness or −b*, blueness; NBS, National Bureau of Standards units; Treat. Int., treatment interaction. ^A,B^ Different capitals indicate significant time-related differences (*p* < 0.05). ^a,b,c^ Different letters indicate differences among treatments (*p* < 0.05).

**Table 7:** Piglets’ faecal volatile compound profile (% of the total volatile compounds).

Volatile Compound	Piglet Groups						*p*								
	C-I 21 Day	TG-II 21 Day	TG-III 21 Day	C-I 62 Day	TG-II 62 Day	TG-III 62 Day	C-I 21 Day vs. C-I 62 Day	TG-II 21 Day vs. TG-II 62 Day	TG-III 21 Day vs. TG-III 62 Day	C-I 21 Day vs. TG-II 21 Day	C-I 21 Day vs. TG-III 21 Day	TG-II 21 Day vs. TG-III 21 Day	C-I 62 Day. vs. TG-II 62 Day	C-I 62 Day vs. TG-III 62 Day	TG-II 62 Day vs. TG-III 62 Day
Butanoic acid	nd	nd	0.374	9.46	13.11	24.14	-	-	<0.001	-	-	-	0.003	<0.001	0.001
Butanoic acid, 3-methyl-	nd	nd	nd	nd	0.877	1.42	-	-	-	-	-	-	-	-	0.006
Butyric acid (2-methyl-)	nd	nd	nd	nd	1.93	2.97	-	-	-	-	-	-	-	-	0.004
Butanoic acid, 2-methyl-	nd	nd	nd	8.07	nd	nd	-	-	-	-	-	-	-	-	-
Pentanoic acid	nd	1.55	0.145	12.64	15.91	24.67	-	0.002	0.007	-	-	0.002	0.004	0.016	0.021
Benzaldehyde	2.22	1.36	4.34	nd	nd	nd	-	-	-	0.031	0.006	<0.001	-	-	-
Trisulfide (dimethyl-)	0.422	nd	3.55	0.095	nd	nd	<0.001	-	-	-	0.002	-	-	-	-
Phosphonic acid, (p-hydroxyphenyl)-	nd	nd	3.33	nd	nd	nd	-	-	-	-	-	-	-	-	-
Phenol	9.4	nd	nd	nd	nd	nd	-	-	-	-	-	-	-	-	-
Isothiocyanate (3-butenyl-)	nd	nd	nd	nd	7.6	1.94	-	-	-	-	-	-	-	-	<0.001
Hexanoic acid	nd	nd	nd	2.73	nd	3.93	-	-	-	-	-	-	-	0.005	-
p-Cresol	56.98	66.11	49.74	39.96	40.34	25.53	0.006	0.001	<0.001	0.007	0.002	<0.001	0.264	0.002	0.004
Benzene, 1,3-bis(1,1-dimethylethyl)-	nd	1.07	0.881	0.41	0.165	0.235	-	<0.001	0.007	-	-	0.062	<0.001	<0.001	<0.001
Indole	17.61	10.72	18.14	4.60	5.39	1.21	0.004	<0.001	0.004	0.012	0.043	0.014	0.005	0.001	0.002
1H-Indole, 3-methyl-	2.73	10.45	8.2	13.53	7.47	7.19	0.004	<0.001	0.009	0.002	<0.001	0.009	0.002	0.01	0.32
Butylated Hydroxytoluene	2.06	2.34	0.927	0.896	0.278	0.257	0.008	0.005	0.003	0.013	0.003	0.004	<0.001	<0.001	0.26
n-Nonadecanol-1	1.62	0.500	1.43	0.116	0.089	0.049	<0.001	<0.001	0.004	<0.001	0.08	0.009	<0.001	<0.001	<0.001

(i) A basal diet (C-I—control group); (ii) a basal diet with extruded–fermented wheat bran addition (TG-II—treatment group II); (iii) a basal diet—sugar beet pulps, dried (TG-III—treatment group III). The data are presented by the means (*n* = 10/group). Next, 21 day—the beginning of the experiment; 62 day—the end of experiment. Results were interpreted as significantly different when *p* < 0.05.

**Table 8:** Correlations between piglets’ faecal volatile compounds and other micro-organisms.

Volatile Compound	Parameters of the Piglets’ Faeces									
	TCE		LAB		TCM		*Enterococcus faecalis*		M/Y	
	Pearson Correlation (r) and Significance (p)									
	r	*p*	r	*p*	r	*p*	r	*p*	r	*p*
Butanoic acid	−0.307	0.215	0.666 **	0.003	0.059	0.816	0.502 *	0.034	0.089	0.725
Butanoic acid, 3-methyl-	−0.439	0.068	0.671 **	0.002	−0.114	0.654	0.330	0.181	0.130	0.607
Butyric acid (2-methyl-)	−0.444	0.065	0.680 **	0.002	−0.117	0.645	0.331	0.180	0.131	0.604
Butanoic acid, 2-methyl-	0.357	0.146	−0.026	0.919	0.349	0.155	0.370	0.130	−0.112	0.659
Pentanoic acid	−0.282	0.257	0.703 **	0.001	0.047	0.853	0.533 *	0.023	0.087	0.732
Benzaldehyde	0.409	0.092	−0.546 *	0.019	0.200	0.425	−0.320	0.195	0.282	0.257
Trisulfide (dimethyl-)	0.390	0.110	−0.387	0.113	0.317	0.199	−0.128	0.614	0.347	0.158
Phosphonic acid, (p-hydroxyphenyl)-	0.349	0.156	−0.327	0.185	0.307	0.216	−0.094	0.710	0.328	0.184
Phenol	0.226	0.368	−0.351	0.153	−0.015	0.953	−0.273	0.274	0.134	0.595
Isothiocyanate (3-butenyl-)	−0.558 *	0.016	0.599 **	0.009	−0.332	0.179	0.086	0.734	−0.035	0.892
Hexanoic acid	0.065	0.799	0.358	0.145	0.298	0.230	0.548 *	0.019	0.082	0.746
p-Cresol	0.304	0.220	−0.352	0.152	−0.014	0.956	−0.288	0.247	0.015	0.952
Benzene, 1,3-bis(1,1-dimethylethyl)-	0.234	0.350	−0.129	0.610	0.163	0.518	0.017	0.948	−0.009	0.972
Indole	0.458	0.056	−0.556 *	0.017	0.168	0.504	−0.349	0.156	0.275	0.270
1H-Indole, 3-methyl-	0.271	0.277	0.273	0.274	0.392	0.107	0.527 *	0.025	−0.025	0.922
Butylated Hydroxytoluene	0.335	0.174	−0.390	0.110	0.012	0.964	−0.257	0.302	−0.021	0.935
n-Nonadecanol-1	0.452	0.060	−0.616 **	0.007	0.173	0.492	−0.374	0.127	0.312	0.207

LAB, lactic acid bacteria count; TCE, total count of enterobacteria; TCM, total count of aerobic and facultative anaerobic micro-organisms; Y/F, yeast/fungi; DM, dry matter. Results were interpreted as significantly different when *p* < 0.05. Blue colour indicates significant correlations; *—statistically significant correlation (*p* < 0.05); **—statistically significant correlation (*p* ≤ 0.0001).

## Data Availability

Not applicable.

## Supplementary Materials

The following supporting information can be downloaded at: https://www.mdpi.com/article/10.3390/ani12091092/s1. Supplementary File S1. Extrusion and fermentation parameters; Supplementary File S2. Piglets’ faecal volatile compounds’ profile.
