# Supplementary material for: Strategy for Local Plant-Based Material Valorisation to Higher-Value Feed Stock for Piglets
Source: Animals (Basel). 2022 Apr 22;12(9):1092. doi: 10.3390/ani12091092 (PMC9100104; doi:10.3390/ani12091092)
Supplement: Supplementary file 1 [file animals-12-01092-s001.zip › Supplementary File S1. Extrusion and Fermentation Parameters.pdf]

Extrusion Parameters

Extrusion of wheat bran was performed at different temperatures in a Twin Screw extruder (Jinan Shengrun Machinery Co., Ltd., Jinan, China). Extrusion parameters are given in **Supplementary file 1**. The temperature in the different extrusion zones was I – 60–61 °C, II – 70 °C, and III – 90 °C; moisture content was 20%, feed rate F was 8.2 ± 0.3 kg h−1, and nozzle diameter was 6 mm. Four different treated wheat bran samples were prepared (Wex115 – extruded at 115 °C with a screw speed of 16 rpm; $W_{ex130/screwspeed16}$ – extruded at 130 °C and 16 rpm; $W_{ex130/screwspeed20}$ – extruded at 130 °C and 20 rpm; $W_{ex130/screwspeed25}$ – extruded at 130 °C and 25 rpm) and non-extruded wheat bran were used as a control (W_{Con} – control wheat bran samples).

Fermentation Parameters

Lactic acid bacteria (LAB) strains *L. casei* and *L. paracasei* (obtained from the Department of Food Safety and Quality at the Lithuanian University of Health Sciences, Kaunas, Lithuania) were used for fermentation of extruded and non-extruded wheat bran.

Before experiments, the LAB strains were stored at −80 °C (Microbank system, Pro-Lab Diagnostics, Birkenhead, UK) and multiplied in MRS broth (de Man–Rogosa–Sharpe, CM 0359, Oxoid Ltd, Hampshire, UK) at 30 ± 2 °C for 48 h, before use for the fermentation of wheat bran.

The wheat bran, water, and a suspension of LAB strain (3% of dry matter of the wheat bran mass) containing 8.9 $\log_{10}$ CFU mL^{-1} were fermented at 30 ± 2 °C for 24 h. For 100 g of wheat bran, 60 mL water was used.

Finally, ten fermented wheat bran samples were prepared (from non-extruded wheat bran: W_{ConLc}, W_{ConLpa}; from extruded at 115 °C with a screw speed of 16 rpm and fermented: $W_{ex115Lc}$, $W_{ex115Lpa}$; from extruded at 130 °C and 16 rpm and fermented: $W_{ex130/screwspeed16Lc}$, $W_{ex130/screwspeed16Lpa}$; from extruded at 130 °C and 20 rpm and fermented: $W_{ex130/screwspeed20Lc}$, $W_{ex130/screwspeed20Lpa}$; from extruded at 130 °C and 25 rpm and fermented: $W_{ex130/screwspeed25Lc}$, $W_{ex130/screwspeed25Lpa}$). The principal scheme of wheat bran pre-treatment is given in **Figure S1**. Physico-chemical and microbiological characteristics of the extruded and fermented wheat bran are reported by Zokaityte et al. (https://doi.org/10.3390/toxins13020163; https://doi.org/10.1016/j.lwt.2021.111498).

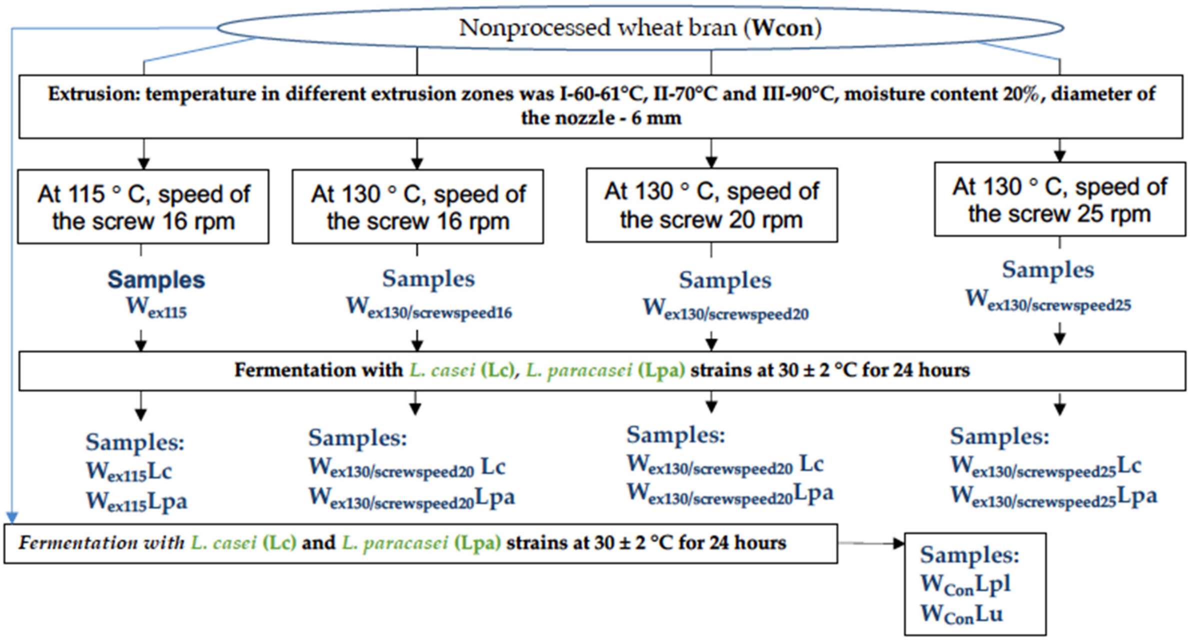

Figure S1. Principal scheme of wheat bran samples treatments.
