# Supplementary material for: Strategy for Local Plant-Based Material Valorisation to Higher-Value Feed Stock for Piglets
Source: Animals (Basel). 2022 Apr 22;12(9):1092. doi: 10.3390/ani12091092 (PMC9100104; doi:10.3390/ani12091092)
Supplement: Supplementary file 1 [file animals-12-01092-s001.zip › Supplementary File S2. Piglets feces volatile compounds profile (1).pdf]

Supplementary File S2. Table S1. Piglets feces volatile compond profile.

Volatile Compound	**Piglets groups**						***p***								
	**C-I 21 d.**	**TG-II 21 d.**	**TG-III 21 d.**	**C-I 62 d.**	**TG-II 62 d.**	**TG-III 62 d.**	**C-I 21d. vs C-I 62d.**	**TG-II 21d. vs TG-II 62d.**	**TG-III 21d. vs TG-III 62d.**	**C-I 21d. vs TG-II .21d.**	**C-I 21d. vs TG-III 21d.**	**TG-II .21d. vs TG-III 21d.**	**C-I 62d. vs TG-II 62d.**	**C-I 62d. vs TG-III 62d.**	**TG-II 62d. vs TG-III 62d.**
Butanoic acid	nd	nd	0.374 ± 0.008	9.46 ± 1.68	13.11 ± 2.19	24.14 ± 1.16	-	-	<.001	-	-	-	0.003	<.001	0.001
Butanoic acid, 3-methyl-	nd	nd	nd	nd	0.877 ± 0.117	1.42 ± 0.22	-	-	-	-	-	-	-	-	0.006
Butyric acid <2-methyl->	nd	nd	nd	nd	1.93 ± 0.3	2.97 ± 0.47	-	-	-	-	-	-	-	-	0.004
Butanoic acid, 2-methyl-	nd	nd	nd	8.07 ± 0.23	nd	nd	-	-	-	-	-	-	-	-	-
Pentanoic acid	nd	1.55 ± 0.19	0.145 ± 0.025	12.64 ± 1.31	15.91 ± 1.82	24.67 ± 5.07	-	0.002	0.007	-	-	0.002	0.004	0.016	0.021
Benzaldehyde	2.22 ± 0.47	1.36 ± 0.08	4.34 ± 0.08	nd	nd	nd	-	-	-	0.031	0.006	<.001	-	-	-
Pentanoic acid, 4-methyl-	nd	nd	nd	nd	nd	0.052 ± 0.007	-	-	-	-	-	-	-	-	-
Trisulfide <dimethyl->	0.422 ± 0.018	nd	3.55 ± 0.32	0.095 ± 0.008	nd	nd	<.001	-	-	-	0.002	-	-	-	-
Phenyl alcohol	nd	0.589 ± 0.101	nd	nd	nd	nd	-	-	-	-	-	-	-	-	-
Phosphonic acid, (p-hydroxyphenyl)-	nd	nd	3.33 ± 0.31	nd	nd	nd	-	-	-	-	-	-	-	-	-
Phenol	9.4 ± 2.03	nd	nd	nd	nd	nd	-	-	-	-	-	-	-	-	-
Isothiocyanate <3-butenyl->	nd	nd	nd	nd	7.6 ± 0.72	1.94 ± 0.38	-	-	-	-	-	-	-	-	<.001
Propyl valerate	nd	nd	nd	nd	0.54 ± 0.061	nd	-	-	-	-	-	-	-	-	-
Hexanoic acid	nd	nd	nd	2.73 ± 0.16	nd	3.93 ± 0.36	-	-	-	-	-	-	-	0.005	-
2-Propyl-1-pentanol	nd	nd	0.07 ± 0.01	nd	nd	nd	-	-	-	-	-	-	-	-	-
Hexanol <2-ethyl->	0.086 ± 0.006	nd	nd	nd	nd	nd	-	-	-	-	-	-	-	-	-
Benzyl alcohol	nd	nd	0.664 ± 0.06	0.094 ± 0.004	0.049 ± 0.011	nd	-	-	-	-	-	-	0.004	-	-

Benzeneacetaldehyde	nd	0.198 ± 0.042	0.255 ± 0.047	0.114 ± 0.015	0.188 ± 0.043	0.158 ± 0.033	-	0.002	0.003	-	-	0.001	0.022	0.026	0.018
(R)-(-)-4-Methylhexanoic acid	nd	nd	nd	nd	nd	0.023 ± 0.004	-	-	-	-	-	-	-	-	-
2-Octenal, (E)-	nd	nd	0.095 ± 0.017	nd	nd	nd	-	-	-	-	-	-	-	-	-
n-Butyric acid 2-ethylhexyl ester	nd	nd	nd	0.09 ± 0.012	nd	0.111 ± 0.016	-	-	-	-	-	-	-	0.006	-
Acetophenone	nd	nd	nd	nd	0.036 ± 0.003	0.056 ± 0.013	-	-	-	-	-	-	-	-	0.037
6-Benzoylhexanoic acid	nd	nd	nd	0.052 ± 0.008	nd	nd	-	-	-	-	-	-	-	-	-
p-Cresol	56.98 ± 5.38	66.11 ± 3.44	49.74 ± 4.5	39.96 ± 2.16	40.34 ± 1.29	25.53 ± 3.59	0.006	0.001	<.001	0.007	0.002	<.001	0.264	0.002	0.004
Phenol, 2-methoxy-	0.71 ± 0.1	nd	nd	nd	nd	nd	-	-	-	-	-	-	-	-	-
Butanoic acid, 3-methyl-, butyl ester	nd	nd	nd	nd	0.419 ± 0.088	nd	-	-	-	-	-	-	-	-	-
2-Nonanone	nd	0.164 ± 0.009	0.616 ± 0.11	0.311 ± 0.055	nd	nd	-	-	-	-	-	0.008	-	-	-
2-Nonanol	nd	nd	nd	nd	nd	0.06 ± 0.012	-	-	-	-	-	-	-	-	-
1,6-Octadien-3-ol, 3,7-dimethyl-	0.103 ± 0.02	nd	0.134 ± 0.011	0.149 ± 0.006	0.413 ± 0.049	nd	0.015	-	-	-	0.013	-	0.004	-	-
Nonanal	0.142 ± 0.022	0.145 ± 0.02	0.175 ± 0.004	0.056 ± 0.006	nd	nd	0.006	-	-	0.061	0.043	0.042	-	-	-
Phenethyl alcohol	0.666 ± 0.037	0.246 ± 0.038	0.869 ± 0.173	0.548 ± 0.05	0.582 ± 0.041	0.654 ± 0.087	0.002	<.001	0.025	<.001	0.061	0.008	0.011	0.019	0.057
Benzyl methyl ketone	0.043 ± 0.002	0.071 ± 0.007	nd	0.049 ± 0.003	nd	nd	0.005	-	-	0.005	-	-	-	-	-
Bicyclo[3.1.1]heptan-3-ol, 6,6-dimethyl-2-methylene-	0.041 ± 0.004	nd	nd	nd	nd	nd	-	-	-	-	-	-	-	-	-
3-Nonen-1-ol, (Z)-	nd	nd	nd	0.017 ± 0.001	nd	nd	-	-	-	-	-	-	-	-	-
Verbenol <trans->	0.214 ± 0.023	nd	nd	nd	nd	nd	-	-	-	-	-	-	-	-	-
2-Nonenal, (E)-	nd	0.119 ± 0.015	0.05 ± 0.007	0.126 ± 0.019	0.076 ± 0.007	0.037 ± 0.006	-	0.006	<.001	-	-	0.002	0.009	0.004	<.001
Phenol, 4-ethyl-	0.113 ± 0.013	0.462 ± 0.097	0.098 ± 0.022	1.01 ± 0.06	0.084 ± 0.003	nd	<.001	0.01	-	0.009	0.051	0.007	<.001	-	-
Octanoic acid	nd	nd	nd	nd	0.395 ± 0.044	0.665 ± 0.095	-	-	-	-	-	-	-	-	0.006

Cyclopropane, nonyl-	nd	nd	0.089 ± 0.008	nd	nd	nd	-	-	-	-	-	-	-	-	-
1,7,7-Trimethylbicyclo[2.2.1]heptan-2-ol	0.278 ± 0.008	nd	nd	nd	nd	nd	-	-	-	-	-	-	-	-	-
2-Piperidinone	0.162 ± 0.01	0.129 ± 0.026	0.306 ± 0.051	nd	nd	nd	-	-	-	0.035	0.013	0.003	-	-	-
Nonanenitrile	nd	nd	nd	0.02 ± 0.002	nd	nd	-	-	-	-	-	-	-	-	-
2-Decanone	nd	0.076 ± 0.008	0.071 ± 0.012	0.151 ± 0.005	0.117 ± 0.022	0.089 ± 0.005	-	0.018	0.023	-	-	0.081	0.037	<.001	0.052
Creosol	nd	nd	nd	nd	0.049 ± 0.002	nd	-	-	-	-	-	-	-	-	-
L-.alpha.-Terpineol	0.574 ± 0.072	nd	0.103 ± 0.003	nd	nd	nd	-	-	-	-	0.004	-	-	-	-
Octanoic acid, ethyl ester	nd	nd	nd	0.016 ± 0.002	nd	0.021 ± 0.003	-	-	-	-	-	-	-	0.007	-
Methoxyacetic acid, 2-tetradecyl ester	nd	nd	nd	nd	nd	0.082 ± 0.009	-	-	-	-	-	-	-	-	-
Dodecane	0.142 ± 0.004	0.187 ± 0.035	0.224 ± 0.02	0.117 ± 0.014	nd	nd	0.025	-	-	0.064	0.006	0.025	-	-	-
Decanal	nd	0.047 ± 0.008	0.039 ± 0.002	nd	nd	nd	-	-	-	-	-	0.074	-	-	-
Tetrasulfide <methyl->	0.185 ± 0.018	nd	0.781 ± 0.116	nd	0.044 ± 0.003	nd	-	-	-	-	0.004	-	-	-	-
Benzothiazole	nd	nd	nd	0.089 ± 0.009	0.03 ± 0.002	nd	-	-	-	-	-	-	0.002	-	-
3-Phenylpropanol	nd	nd	nd	nd	0.071 ± 0.013	0.053 ± 0.007	-	-	-	-	-	-	-	-	0.018
3,6-Octadien-1-ol, 3,7-dimethyl-, (Z)-	0.411 ± 0.094	nd	nd	nd	nd	nd	-	-	-	-	-	-	-	-	-
Quinoline	nd	nd	nd	nd	0.019 ± 0.001	nd	-	-	-	-	-	-	-	-	-
Benzeneacetic acid	nd	nd	nd	0.023 ± 0.004	nd	0.039 ± 0.006	-	-	-	-	-	-	-	0.003	-
Chavicol	nd	nd	nd	0.037 ± 0.005	0.024 ± 0.002	0.034 ± 0.003	-	-	-	-	-	-	0.009	0.061	0.002
Benzene, 1,3-bis(1,1-dimethylethyl)-	nd	1.07 ± 0.04	0.881 ± 0.167	0.41 ± 0.018	0.165 ± 0.034	0.235 ± 0.031	-	<.001	0.007	-	-	0.062	<.001	<.001	<.001
Nonanoic acid	nd	nd	nd	0.292 ± 0.045	0.201 ± 0.014	0.241 ± 0.017	-	-	-	-	-	-	0.018	0.044	<.001
Acetic acid, trifluoro-, nonyl ester	nd	nd	nd	0.023 ± 0.001	nd	nd	-	-	-	-	-	-	-	-	-

Phenol, 3-methyl-4-(methylthio)-	0.117 ± 0.025	nd	nd	nd	0.191 ± 0.019	0.058 ± 0.006	-	-	-	-	-	-	-	-	0.002
Dodecane, 4,6-dimethyl-	nd	nd	nd	0.046 ± 0.007	0.045 ± 0.003	0.026 ± 0.001	-	-	-	-	-	-	0.354	0.014	0.002
Guaiacol <4-ethyl->	0.106 ± 0.006	nd	nd	nd	nd	nd	-	-	-	-	-	-	-	-	-
2-Undecanone	0.378 ± 0.082	0.214 ± 0.023	0.306 ± 0.037	0.199 ± 0.045	0.217 ± 0.009	0.128 ± 0.005	0.007	0.373	0.005	0.02	0.055	0.004	0.239	0.046	<.001
Indole	17.61 ± 2.41	10.72 ± 0.49	18.14 ± 2.7	4.6 ± 0.44	5.39 ± 0.58	1.21 ± 0.14	0.004	<.001	0.004	0.012	0.043	0.014	0.005	0.001	0.002
Ethanone, 1-(2-aminophenyl)-	nd	nd	0.07 ± 0.004	nd	nd	nd	-	-	-	-	-	-	-	-	-
Hydrocinnamic acid	nd	nd	nd	nd	0.076 ± 0.011	0.128 ± 0.022	-	-	-	-	-	-	-	-	0.007
Hydrocinnamate <ethyl->	nd	nd	nd	0.008 ± 0.001	0.014 ± 0	nd	-	-	-	-	-	-	0.005	-	-
Decyl methyl ketone	nd	0.063 ± 0.009	0.061 ± 0.011	0.048 ± 0.004	nd	nd	-	-	-	-	-	0.113	-	-	-
Propanoic acid, 2-methyl-, 2,2-dimethyl-1-(2-hydroxy-1-methylethyl)propylester	0.029 ± 0.006	nd	nd	nd	nd	nd	-	-	-	-	-	-	-	-	-
n-Decanoic acid	nd	nd	nd	0.186 ± 0.033	0.144 ± 0.015	0.319 ± 0.046	-	-	-	-	-	-	0.028	0.002	0.005
3-Allyl-6-methoxyphenol	0.237 ± 0.026	nd	nd	nd	nd	nd	-	-	-	-	-	-	-	-	-
2(3H)-Furanone, dihydro-5-pentyl-	nd	nd	nd	0.305 ± 0.048	nd	0.145 ± 0.016	-	-	-	-	-	-	-	0.007	-
2(3H)-Furanone, 5-heptyldihydro-	nd	nd	nd	nd	0.172 ± 0.033	nd	-	-	-	-	-	-	-	-	-
.gamma.-Dodecalactone	nd	0.119 ± 0.009	nd	nd	nd	nd	-	-	-	-	-	-	-	-	-
Ionol <alpha->	0.041 ± 0.001	nd	nd	nd	nd	nd	-	-	-	-	-	-	-	-	-
1H-Indole, 3-methyl-	2.73 ± 0.39	10.45 ± 1.19	8.2 ± 0.66	13.53 ± 2.03	7.47 ± 1.31	7.19 ± 0.42	0.004	<.001	0.009	0.002	<.001	0.009	0.002	0.01	0.32
Tetradecane	0.158 ± 0.008	0.144 ± 0.016	0.175 ± 0.023	0.203 ± 0.032	0.124 ± 0.012	0.132 ± 0.014	0.042	0.007	0.007	0.047	0.094	0.008	0.01	0.01	0.01
Dodecanal	0.069 ± 0.002	0.052 ± 0.01	0.047 ± 0.01	0.04 ± 0.005	0.014 ± 0.002	0.02 ± 0.002	0.002	0.007	0.014	0.033	0.021	<.001	0.002	0.004	<.001
3-Buten-2-one, 4-(2,6,6-trimethyl-2-cyclohexen-1-yl)-	0.04 ± 0.002	nd	nd	nd	nd	nd	-	-	-	-	-	-	-	-	-

Isothiocyanate <4-(methylthio)butyl->	nd	nd	nd	nd	0.037 ± 0.007	0.013 ± 0.001	-	-	-	-	-	-	-	-	0.01
5,9,13-Pentadecatrien-2-one, 6,10,14-trimethyl-, (E,E)-	nd	nd	nd	nd	0.053 ± 0.007	0.041 ± 0.004	-	-	-	-	-	-	-	-	0.01
2,6,10-Dodecatrien-1-ol, 3,7,11-trimethyl-	nd	nd	nd	0.05 ± 0.008	nd	nd	-	-	-	-	-	-	-	-	-
5,9-Undecadien-2-one, 6,10-dimethyl-, (Z)-	0.068 ± 0.006	nd	0.123 ± 0.01	nd	nd	nd	-	-	-	-	<.001	-	-	-	-
5,9-Undecadien-2-one, 6,10-dimethyl-	nd	0.044 ± 0.009	nd	nd	nd	nd	-	-	-	-	-	-	-	-	-
Hexadecane, 2,6,10,14-tetramethyl-	nd	nd	nd	0.035 ± 0.004	nd	nd	-	-	-	-	-	-	-	-	-
7,11-Dimethyldodeca-2,6,10-trien-1-ol	nd	nd	nd	0.179 ± 0.019	0.124 ± 0.009	0.117 ± 0.017	-	-	-	-	-	-	0.005	<.001	0.134
Supraene	nd	nd	0.129 ± 0.014	nd	nd	nd	-	-	-	-	-	-	-	-	-
3,7,11-Tridecatrienenitrile, 4,8,12-trimethyl-	nd	0.072 ± 0.003	nd	nd	nd	nd	-	-	-	-	-	-	-	-	-
Hexadecane, 1,1-bis(dodecyloxy)-	nd	nd	nd	0.043 ± 0.002	nd	nd	-	-	-	-	-	-	-	-	-
Cyclopentane, 1-pentyl-2-propyl-	nd	nd	0.132 ± 0.015	0.23 ± 0.049	0.075 ± 0.003	nd	-	-	-	-	-	-	0.014	-	-
n-Pentadecanol	0.512 ± 0.057	nd	0.474 ± 0.081	0.306 ± 0.044	nd	nd	<.001	-	-	-	0.056	-	-	-	-
Trifluoroacetic acid,n-tridecyl ester	nd	0.261 ± 0.049	nd	nd	nd	0.082 ± 0.006	-	-	-	-	-	-	-	-	-
Tridecanal	nd	0.162 ± 0.014	0.052 ± 0.002	0.056 ± 0.01	0.058 ± 0.008	0.081 ± 0.013	-	<.001	0.022	-	-	0.002	0.113	0.002	0.008
Phenol, 2,4-bis(1,1-dimethylethyl)-	nd	nd	nd	0.085 ± 0.005	nd	nd	-	-	-	-	-	-	-	-	-
Butylated Hydroxytoluene	2.06 ± 0.29	2.34 ± 0.37	0.927 ± 0.143	0.896 ± 0.033	0.278 ± 0.011	0.257 ± 0.058	0.008	0.005	0.003	0.013	0.003	0.004	<.001	<.001	0.26
5-tert-Butylpyrogallol	nd	nd	nd	nd	0.022 ± 0.003	nd	-	-	-	-	-	-	-	-	-
Cyclopentane, 1,2,3,4,5-pentamethyl-	0.033 ± 0.004	nd	0.047 ± 0.006	nd	nd	nd	-	-	-	-	0.003	-	-	-	-
Isoamyl salicylate	nd	nd	nd	0.06 ± 0.013	0.02 ± 0.004	nd	-	-	-	-	-	-	0.008	-	-
Hexadecane, 1-iodo-	nd	0.024 ± 0.002	nd	nd	nd	nd	-	-	-	-	-	-	-	-	-
Dodecanoic acid	nd	nd	nd	nd	nd	0.03 ± 0.004	-	-	-	-	-	-	-	-	-

cyclododecanol	0.056 ± 0.009	0.513 ± 0.045	0.113 ± 0.008	0.164 ± 0.009	nd	0.23 ± 0.016	<.001	-	<.001	0.001	<.001	0.001	-	0.002	-
Hexamethylene diacrylate	nd	nd	0.122 ± 0.011	0.125 ± 0.012	0.087 ± 0.006	0.097 ± 0.019	-	-	0.016	-	-	-	0.004	0.01	0.157
Hexadecane	0.229 ± 0.045	0.22 ± 0.022	0.26 ± 0.009	0.252 ± 0.041	0.202 ± 0.026	0.164 ± 0.019	0.005	0.008	0.002	0.284	0.137	0.017	0.014	0.01	0.006
Dodecane, 1-methoxy-	nd	nd	nd	nd	nd	0.03 ± 0.006	-	-	-	-	-	-	-	-	-
Dichloroacetic acid, 4-hexadecyl ester	0.052 ± 0.008	0.107 ± 0.015	0.211 ± 0.012	0.055 ± 0.006	0.034 ± 0.001	nd	0.061	0.006	-	0.003	<.001	<.001	0.009	-	-
Tetradecanal	0.079 ± 0.001	0.166 ± 0.004	0.179 ± 0.029	0.123 ± 0.018	0.188 ± 0.032	0.228 ± 0.034	0.023	0.153	0.002	<.001	0.013	0.231	0.008	0.004	<.001
Heptadecanal	nd	nd	nd	nd	nd	0.066 ± 0.004	-	-	-	-	-	-	-	-	-
Oleyl alcohol, trifluoroacetate	0.439 ± 0.074	nd	nd	nd	nd	nd	-	-	-	-	-	-	-	-	-
9-Octadecen-1-ol, (Z)-	nd	nd	0.365 ± 0.019	nd	nd	nd	-	-	-	-	-	-	-	-	-
Oxirane, hexadecyl-	nd	0.205 ± 0.015	nd	nd	nd	nd	-	-	-	-	-	-	-	-	-
1-Nonadecene	0.11 ± 0.021	0.064 ± 0.011	0.129 ± 0.024	nd	nd	nd	-	-	-	0.008	0.004	0.007	-	-	-
2-Pentadecanone	nd	0.297 ± 0.024	0.365 ± 0.057	0.236 ± 0.05	0.189 ± 0.024	0.169 ± 0.014	-	<.001	0.008	-	-	0.035	0.044	0.042	0.037
6,10,14-Trimethyl-pentadecan-2-ol	nd	nd	nd	0.031 ± 0.005	nd	nd	-	-	-	-	-	-	-	-	-
Pentadecanal-	nd	nd	nd	0.137 ± 0.006	nd	0.219 ± 0.047	-	-	-	-	-	-	-	0.037	-
Tridecanol <n->	nd	nd	0.028 ± 0.005	nd	nd	nd	-	-	-	-	-	-	-	-	-
2-Hexadecanone	0.075 ± 0.007	0.099 ± 0.017	0.138 ± 0.011	0.04 ± 0.004	0.03 ± 0.003	nd	0.001	0.007	-	0.027	<.001	0.004	0.002	-	-
Octadecane	0.099 ± 0.02	0.129 ± 0.024	0.134 ± 0.019	0.126 ± 0.027	0.175 ± 0.026	0.167 ± 0.034	0.011	<.001	0.031	0.003	<.001	0.113	<.001	0.005	0.113
Hexadecanal	0.116 ± 0.018	0.22 ± 0.028	0.37 ± 0.031	0.164 ± 0.022	0.327 ± 0.032	0.248 ± 0.033	0.001	<.001	<.001	0.002	<.001	<.001	<.001	0.003	<.001
4-Chlorobutyric acid, pentadecyl ester	nd	0.19 ± 0.016	0.143 ± 0.021	nd	nd	nd	-	-	-	-	-	0.002	-	-	-
Benzoic acid, heptyl ester	0.032 ± 0.005	0.054 ± 0.002	0.038 ± 0.006	0.042 ± 0.008	0.039 ± 0.004	nd	0.014	0.003	-	0.003	0.005	0.01	0.162	-	-
1,2-Benzenedicarboxylic acid, bis(2-methylpropyl) ester	nd	nd	nd	0.074 ± 0.003	0.101 ± 0.008	0.135 ± 0.01	-	-	-	-	-	-	0.006	0.002	<.001

n-Nonadecanol-1	1.62 ± 0.08	0.5 ± 0.007	1.43 ± 0.23	0.116 ± 0.005	0.089 ± 0.005	0.049 ± 0.006	<.001	<.001	0.004	<.001	0.08	0.009	<.001	<.001	<.001
7,9-Di-tert-butyl-1-oxaspiro(4,5)deca-6,9-diene-2,8-dione	nd	nd	nd	0.228 ± 0.039	0.42 ± 0.065	0.67 ± 0.091	-	-	-	-	-	-	0.003	0.002	0.002
Dibutyl phthalate	nd	nd	nd	0.158 ± 0.034	0.17 ± 0.007	0.186 ± 0.03	-	-	-	-	-	-	0.261	0.003	0.176
Hexadecanoic acid, ethyl ester	0.019 ± 0.004	nd	0.03 ± 0.002	0.067 ± 0.007	0.104 ± 0.012	0.115 ± 0.012	<.001	-	0.002	-	0.005	-	0.003	0.002	<.001
9-Octadecenal, (Z)-	nd	0.028 ± 0.003	0.032 ± 0.004	nd	0.049 ± 0.008	0.036 ± 0.004	-	0.009	<.001	-	-	0.01	-	-	0.015
Octadecanal	nd	0.026 ± 0.001	0.034 ± 0.007	nd	nd	nd	-	-	-	-	-	0.074	-	-	-
